# Supplementary material for: Elucidating the molecular programming of a nonlinear non-ribosomal peptide synthetase responsible for fungal siderophore biosynthesis
Source: Nat Commun. 2023 May 17;14:2832. doi: 10.1038/s41467-023-38484-8 (PMC10192304; doi:10.1038/s41467-023-38484-8)
Supplement: Supplementary file 1 — Supplementary Information [file 41467_2023_38484_MOESM1_ESM.pdf]

Supplementary Information

Elucidating the molecular programming of a nonlinear nonribosomal peptide synthetase responsible for fungal siderophore biosynthesis

Matthew Jenner,[1,2,†]* Yang Hai,[3,†,‡]* Hong H. Nguyen,[4‡] Munro Passmore,[1] Will Skyrud,[5‡] Junyong Kim,[4] Neil K. Garg,[4] Wenjun Zhang,[5] Rachel R. Ogorzalek Loo[4] and Yi Tang[3]

[1] Department of Chemistry, University of Warwick, Coventry, CV4 7AL, UK.

[2] Warwick Integrative Synthetic Biology Centre (WISB), University of Warwick, Coventry, CV4 7AL, UK.

[3] Department of Chemical and Biomolecular Engineering, University of California, Los Angeles, USA.

[4] Department of Chemistry and Biochemistry, University of California, Los Angeles, USA.

[5] Department of Chemical and Biomolecular Engineering, University of California, Berkeley, USA.

* Corresponding authors: Matthew Jenner (m.jenner@warwick.ac.uk) and Yang Hai (hai@chem.ucsb.edu).

† These authors contributed equally.

‡ Present addresses:

Department of Chemistry and Biochemistry, University of California, Santa Barbara, USA (Y. H.); Transmed Co., Ltd., Ho Chi Minh City, Vietnam (H. H. N.). Arzeda, 3421 Thorndyke Ave W, Seattle, WA 98119, USA (W. S.).

Supplementary Figures

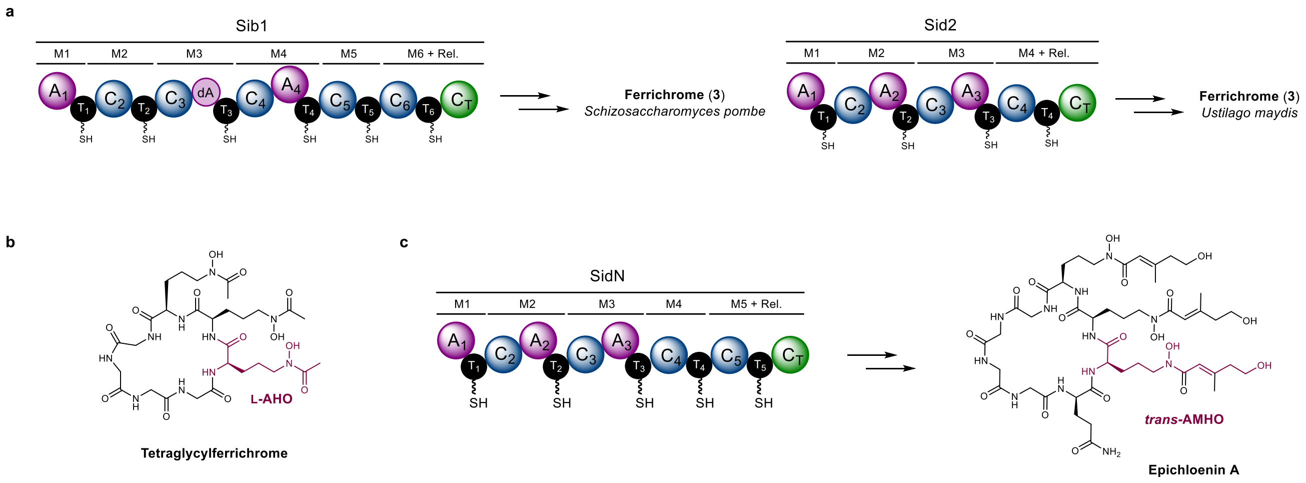

Supplementary Figure 1. Variations on ferrichrome family siderophores from fungi and their biosynthetic origins. **a**). Domain organisation of the Sib1 and Sid2 NRPSs responsible for production of ferrichrome (**3**). Both NRPSs have subtle variations in their domain organisation to each other, yet produce the same peptidyl product. **b**). Structure of tetraglycylferrichrome. **c**). Structure of epichloenin A and domain organisation of the biosynthetic NRPS, SidN.

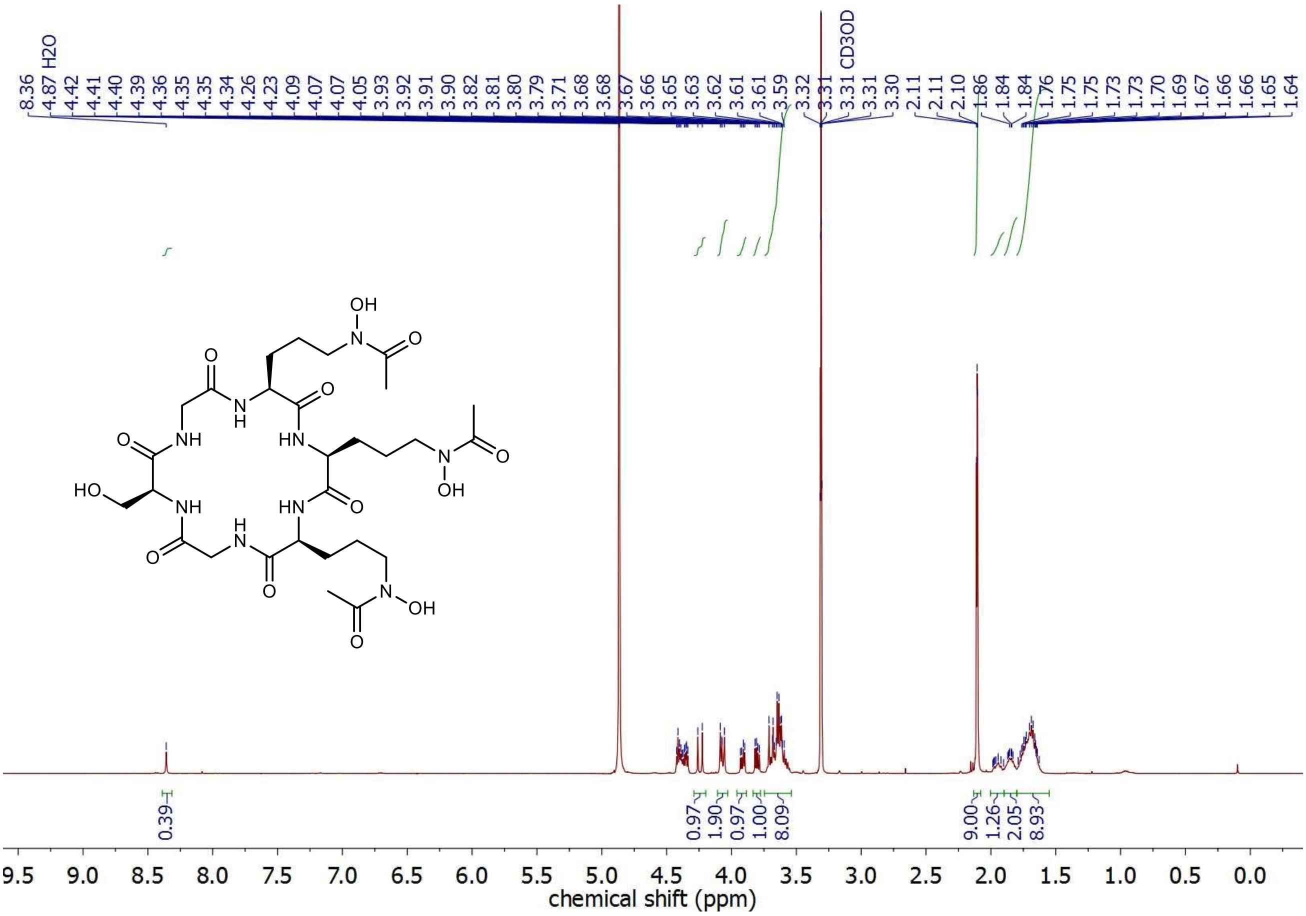

Supplementary Figure 2. ^{1}H NMR spectrum of desferri-ferricrocin in CD_3OD.

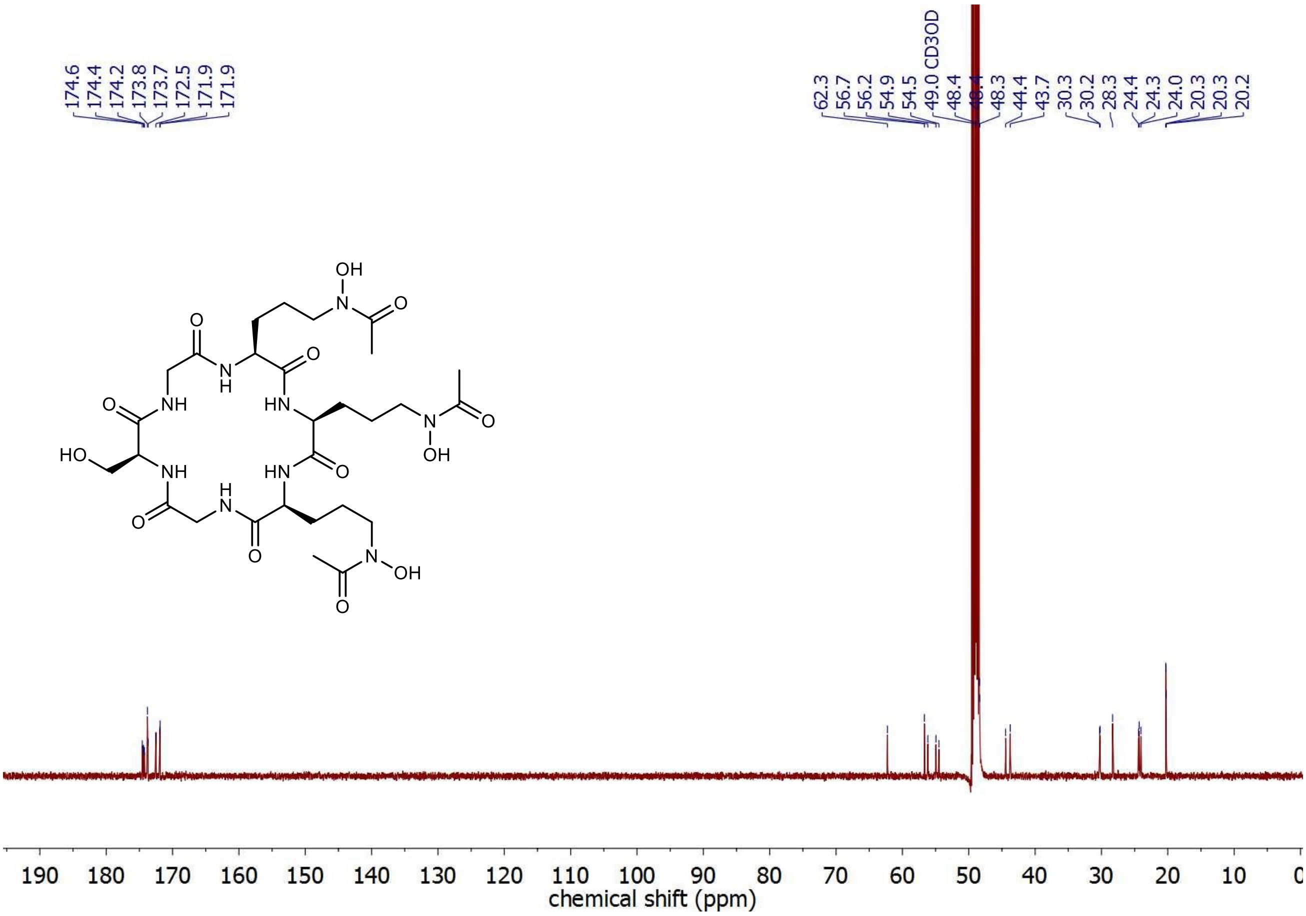

Supplementary Figure 3. ^{13}C NMR spectrum of desferri-ferricrocin in CD_3OD.

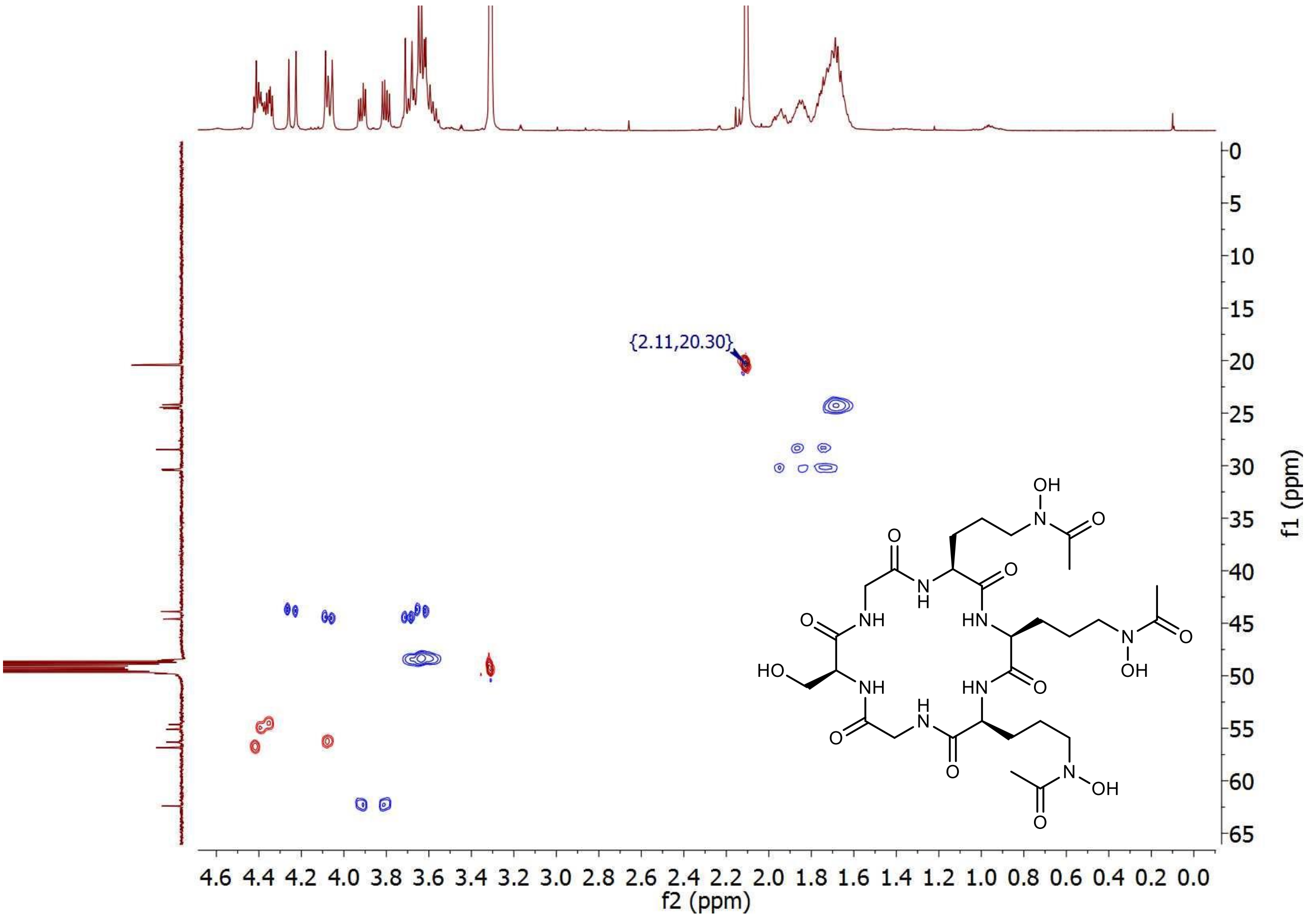

Supplementary Figure 4. HSQC NMR spectrum of desferri-ferricrocin in CD_3OD.

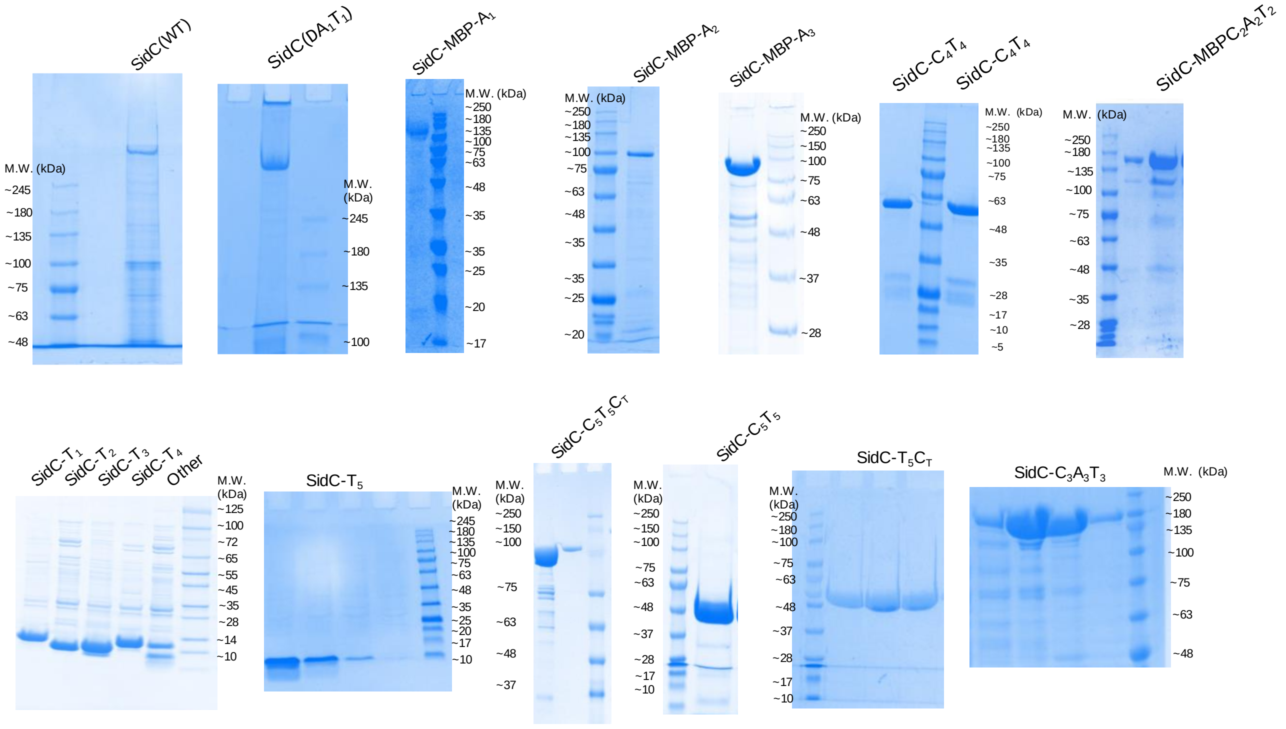

Supplementary Figure 5. SDS-PAGE analysis of recombinant SidC constructs used in this study.

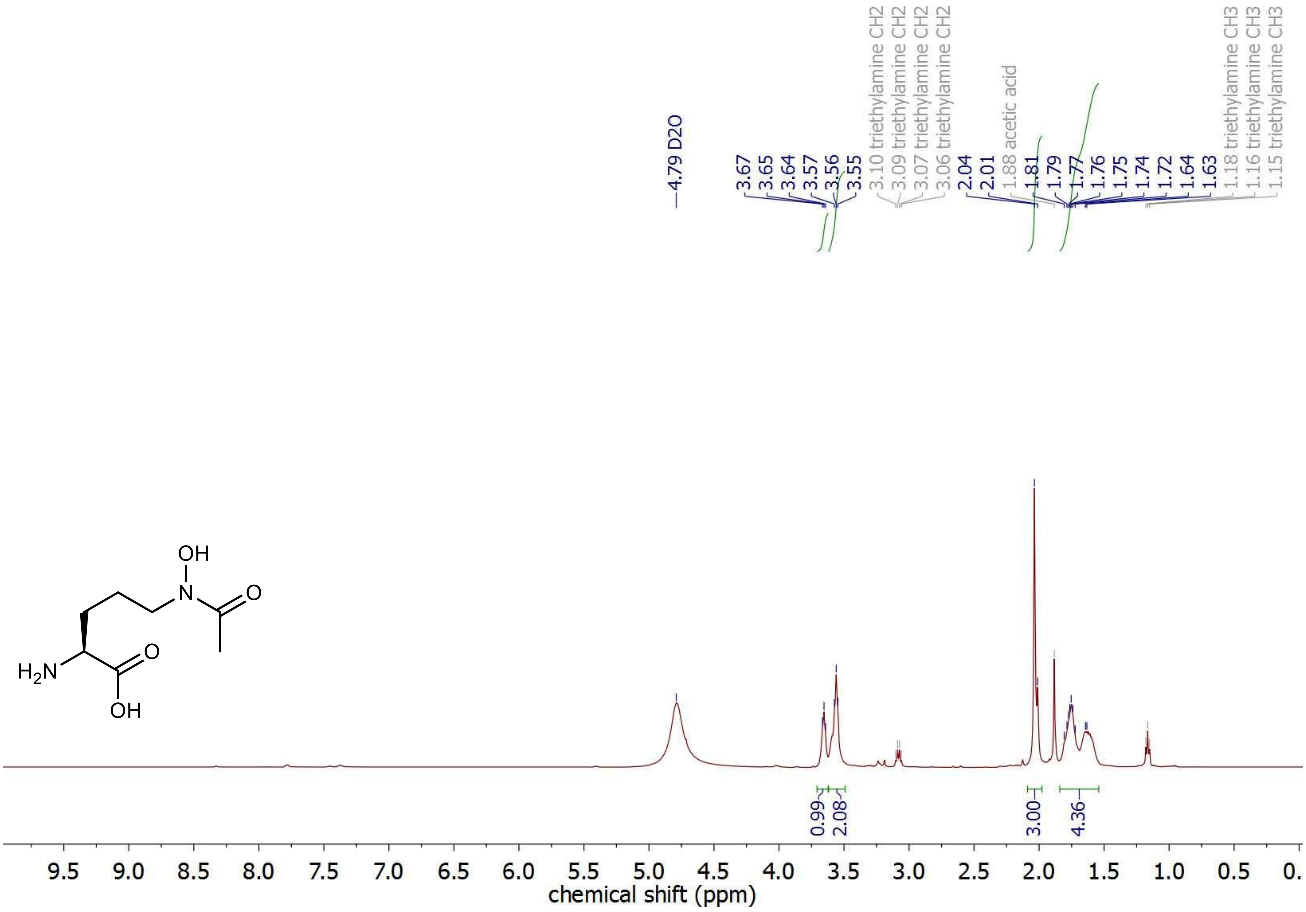

Supplementary Figure 6. ^{1}H-NMR spectrum of L-AHO in D_2O

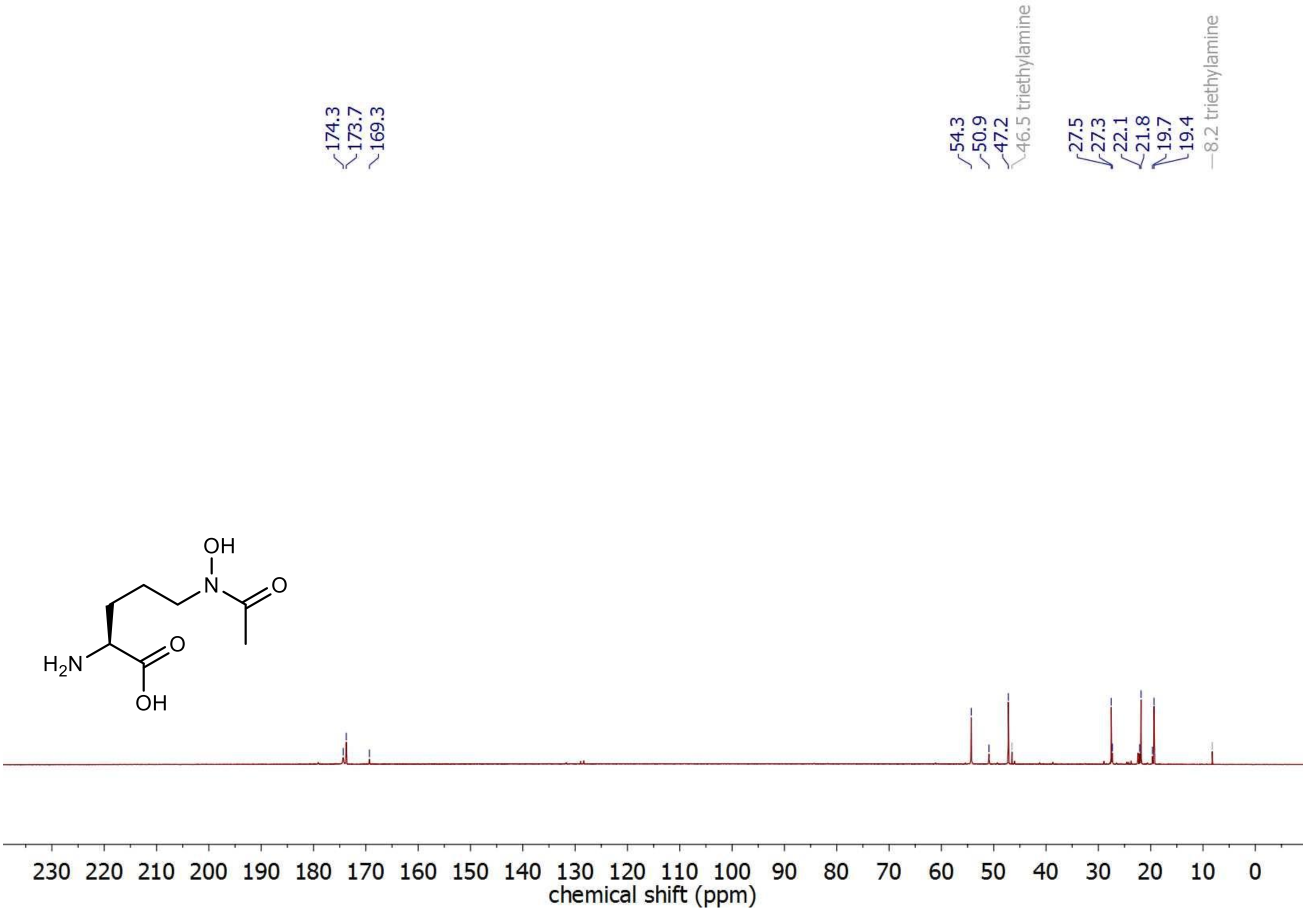

Supplementary Figure 7. ^{13}C-NMR spectrum of L-AHO in D_2O.

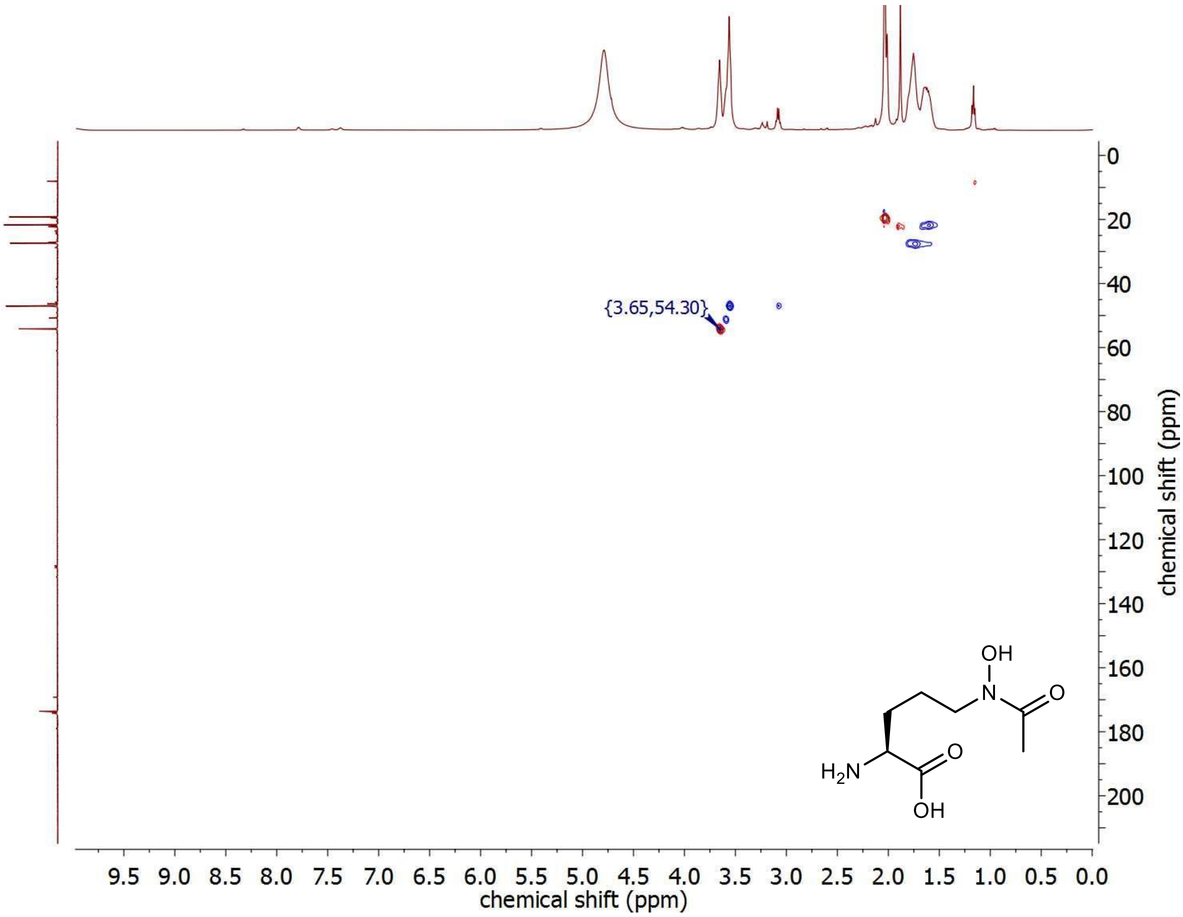

Supplementary Figure 8. HSQC-NMR spectrum of L-AHO in D_2O.

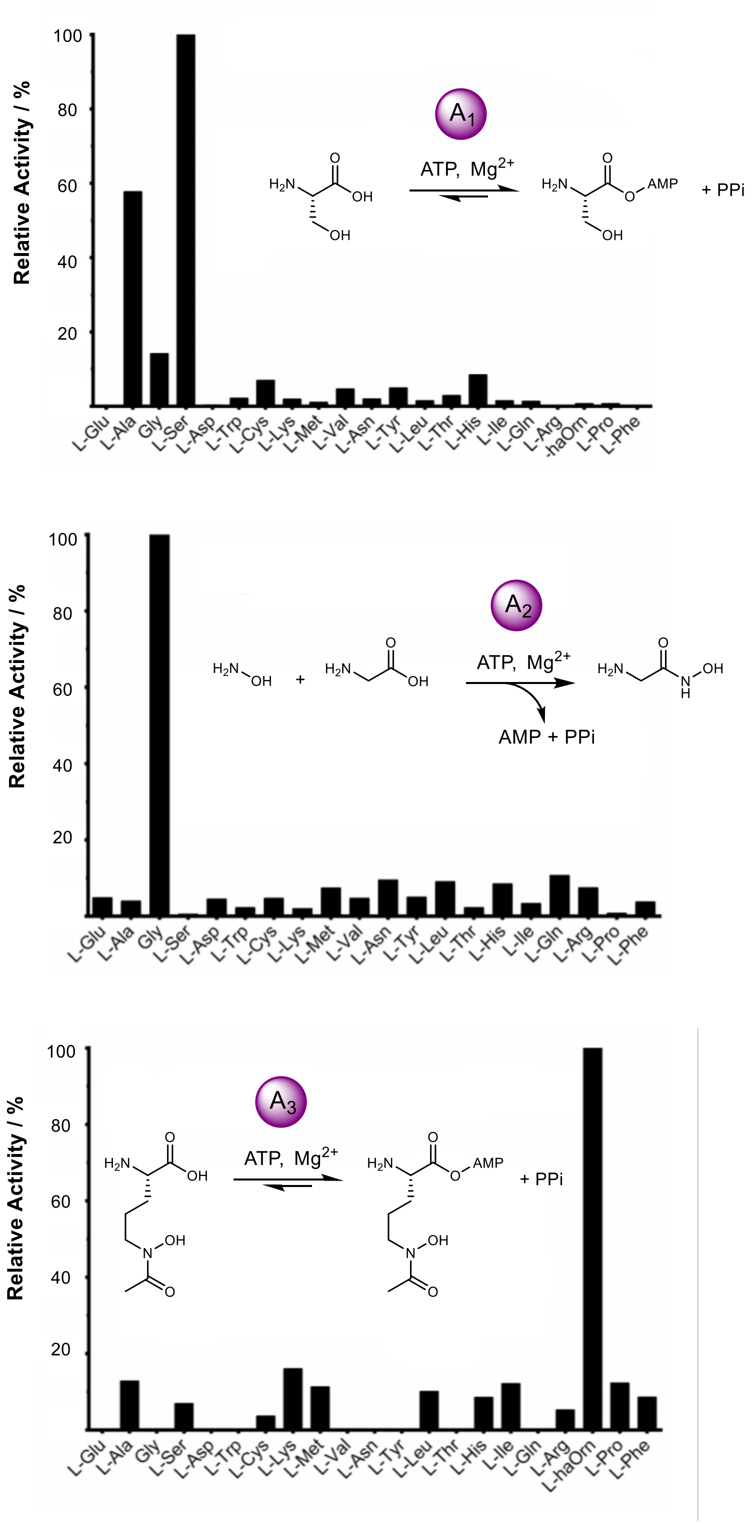

Supplementary Figure 9: Determination of SidC adenylation domain specificity. Analysis of SidC adenylation domain activity *in vitro* using ATP-[^{32}P]PPi exchange assay (A_1 and A_3 domains) and hydroxylamine release assay (A_2 domain) to determine substrate specificity. All values are displayed as relative activity normalised to: L-Ser (*top*, A_1 domain assay); Gly (*middle*, A_2 domain assay); L-AHO (*bottom)*, A_3 domain assay. The constructs and conditions used for each assay are shown with each plot. Experiments were performed as single data point observations for each substrate.

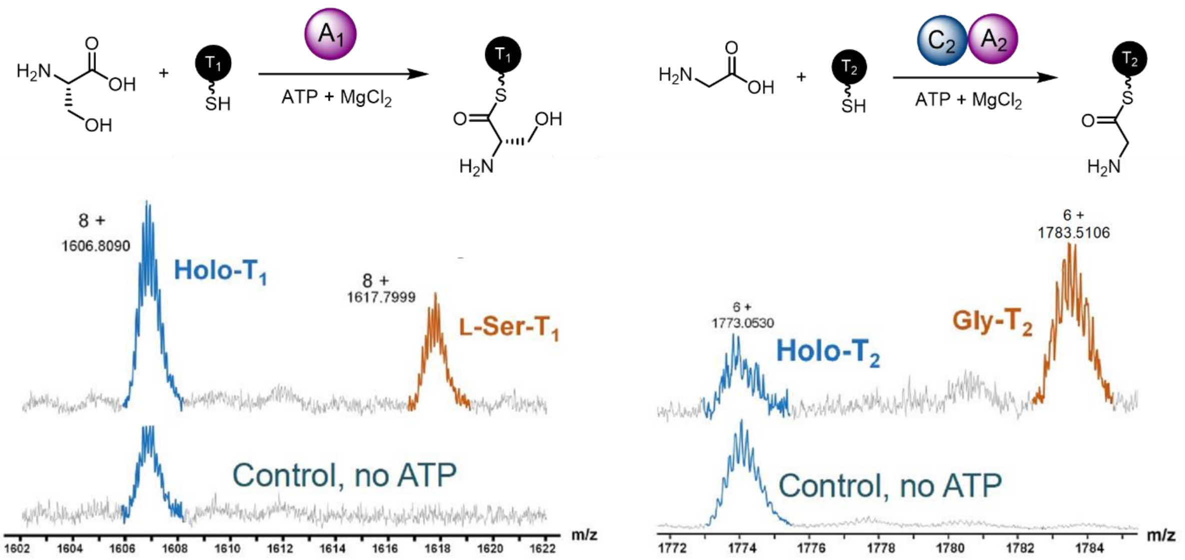

Supplementary Figure 10. Monitoring thiolation step of SidC A_1 and A_2 domains. Intact protein mass spectra of the SidC T_1 (left) and T_2 (right) domains following incubation with their cognate A domain, ATP, Mg^{2+} and the requisite amino acid unit. The 8^+ charge state of the T_1 domain is shown, with *holo*- and L-Ser loaded species highlighted in blue and orange, respectively. The 6^+ charge state of the T_2 domain is shown, with *holo*- and Gly loaded species highlighted in blue and orange, respectively.

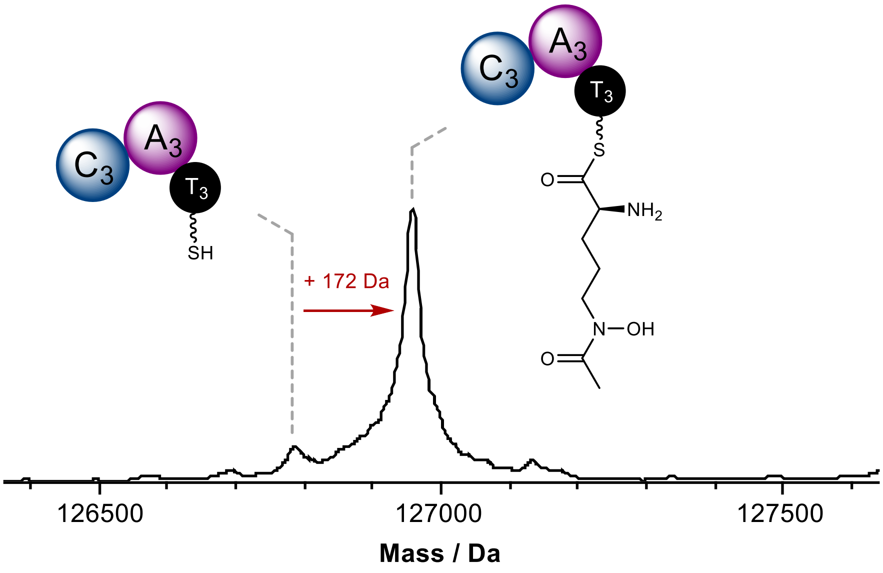

Supplementary Figure 11. SidC A_3 domain loads L-haOrn to the T_3 domain. Deconvoluted intact protein mass spectra of *holo*-SidC $C_3A_3T_3$ following incubation with L-haOrn, ATP and Mg^{2+}, showing loading of a single L-AHO unit onto the T_3 domain, demonstrated by a +172 Da mass shift to the protein. Exact measured and observed masses are detailed in Table S2. Experiment was performed in duplicate and a representative spectrum is shown.

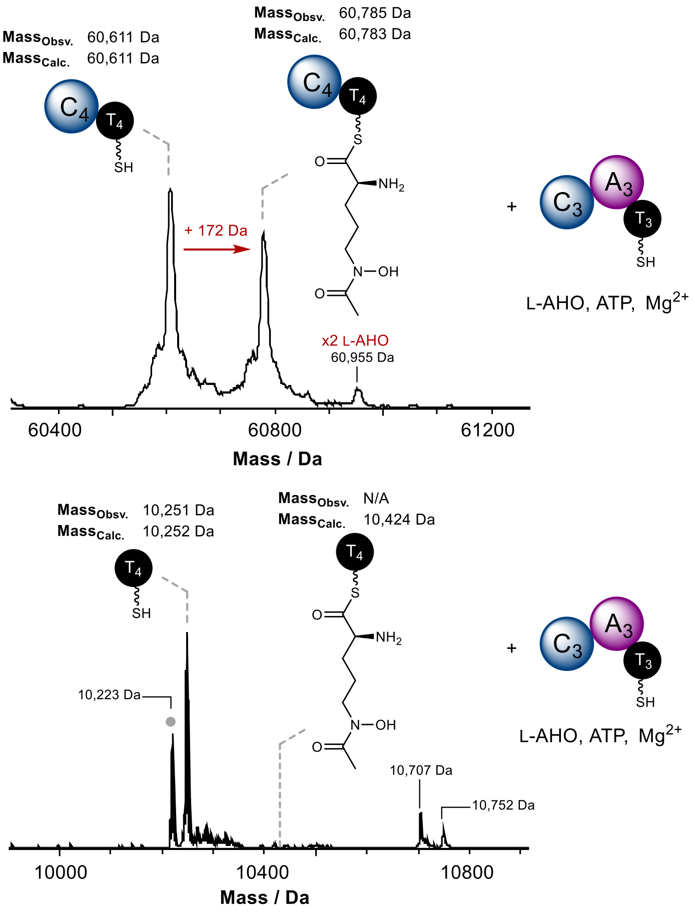

Supplementary Figure 12. Loading of L-AHO by SidC A_3 domain to the T_4 domain requires the upstream C domain. Deconvoluted intact protein mass spectra of *holo*-SidC C_4T_4 (*top*) and *holo*-SidC T_4 (*bottom*) following incubation with *holo*-SidC $C_3A_3T_3$, L-AHO, ATP and Mg^{2+}. Loading of L-AHO is only observed when the N-terminal C domain of each construct is present. Mass shifts corresponding to biosynthetic steps are highlighted with red arrows, and proposed intermediates are displayed. The grey dot indicates a species -28 Da less than the expected mass of SidC T_4. The peak at 60,955 Da suggests trace amount of 2 x L-AHO forming from condensation. Exact measured and observed masses are detailed in the spectra and Table S2. Experiments were performed in duplicate and representative data are shown.

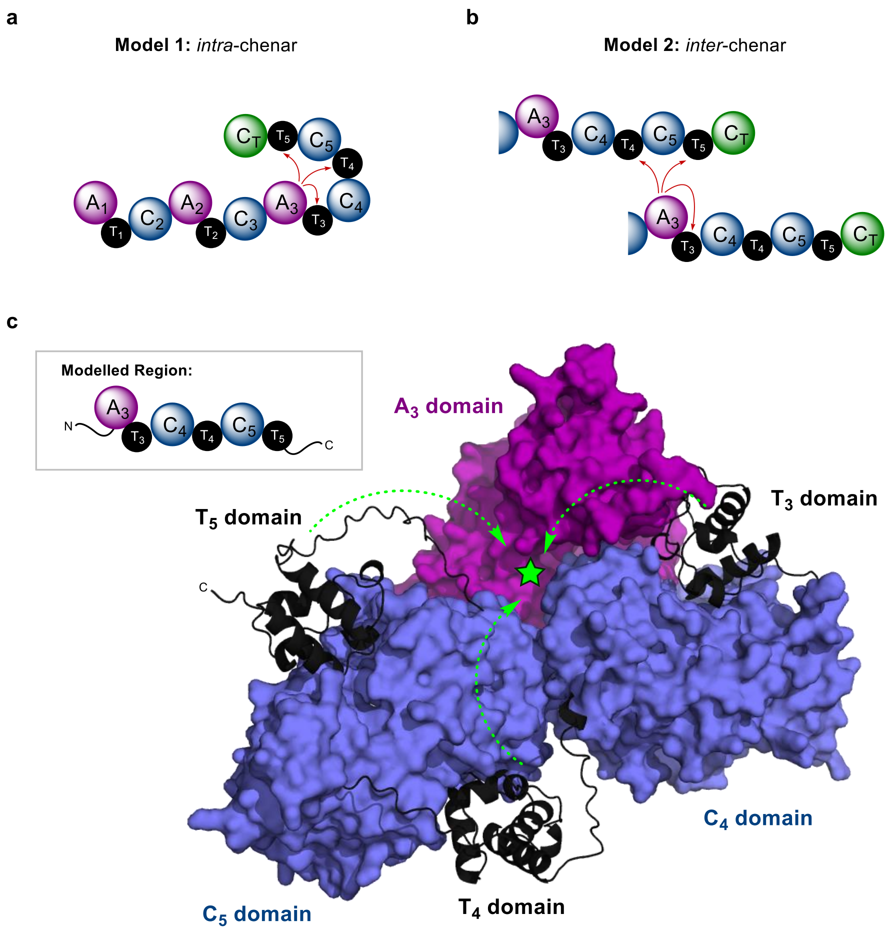

Supplementary Figure 13. Two architectural models for L-AHO by the A3 domain and modelling of the $A_3T_3C_4T_4C_5T_5$ region. a). In model 1, intra-chenar L-AHO loading is facilitated by a 3-dimensional configuration that enables proximity of the A_3 domain to the T_4 and T_5 domains. b). In model 2, the juxtaposition of two SidC proteins allows the A_3 domain to load L-AHO onto the T_4 and T_5 domain in an inter-chenar manner, whilst loading the T_3 domain conventionally. c). AlphaFold model of the $A_3T_3C_4T_4C_5T_5$ region of SidC. The C_4 and C_5 domains both engage in an interface with the A_3 domain, possibly providing a platform for the T domains to access the A_3 domain active site. The opening to the A_3 domain active site is highlighted with a green star, and trajectories of each T domain are shown with green arrows. A co-ordinate file for the SidC $A_3T_3C_4T_4C_5T_5$ region is available for download from Mendeley Data DOI: 10.17632/c3ymyp3yx4.1.

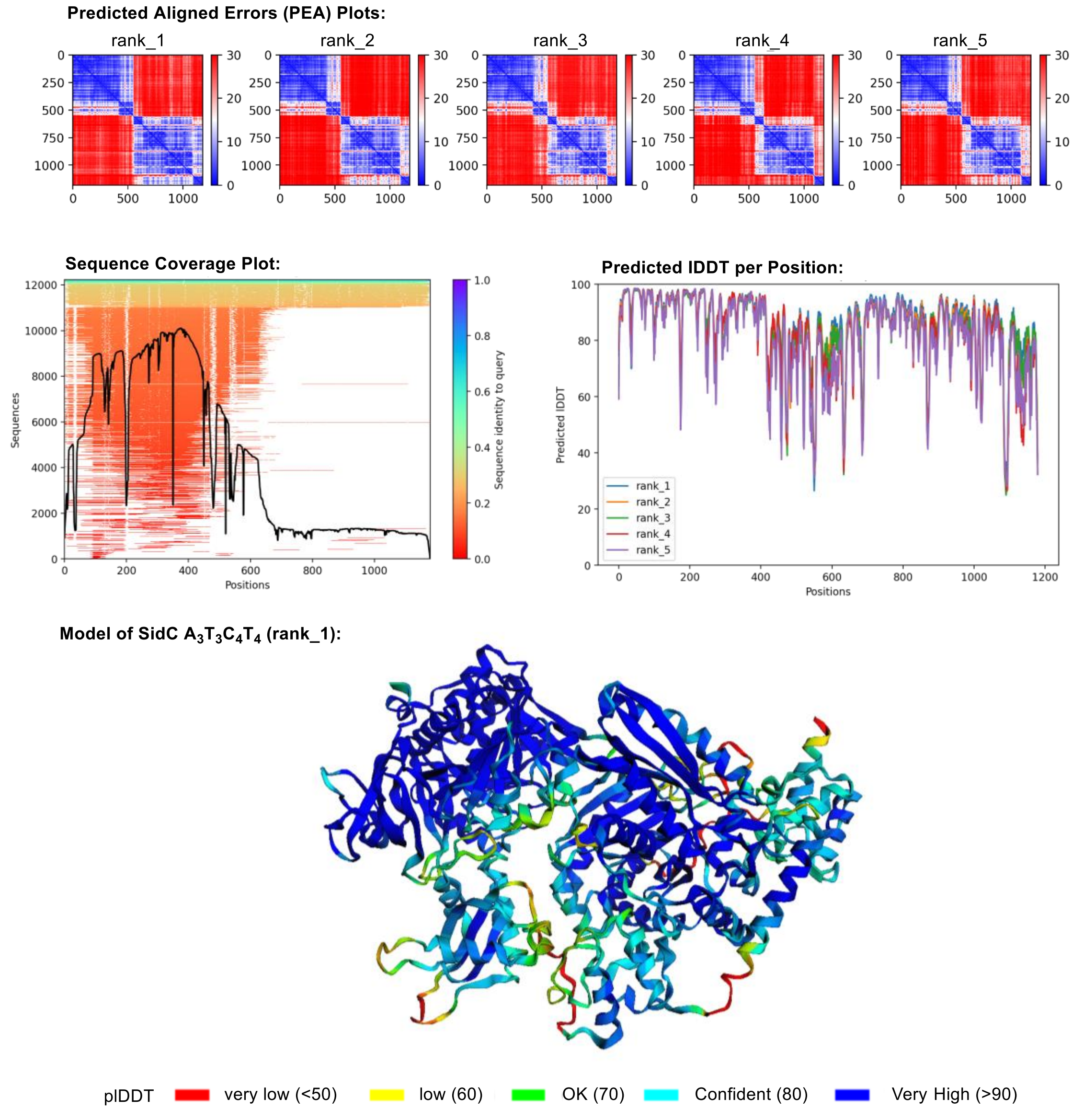

Supplementary Figure 14. Structural assessment of AlphaFold model for SidC $A_3T_3C_4T_4$ fragment. Predicted aligned errors (PEA) plots for the top five ranked models are shown, in addition to sequence coverage and predicted local distance difference test (plDDT) plots. pLDDT values are visualized on the rank_1 structure of SidC $A_3T_3C_4T_4$. The core catalytic domains have pLDDT scores >80, whereas linker regions between domains have scores <50 consistent with previous observations of their disordered nature.[1] A co-ordinate file for the SidC $A_3T_3C_4T_4$ region is available for download from Mendeley Data DOI: 10.17632/c3ymyp3yx4.1.

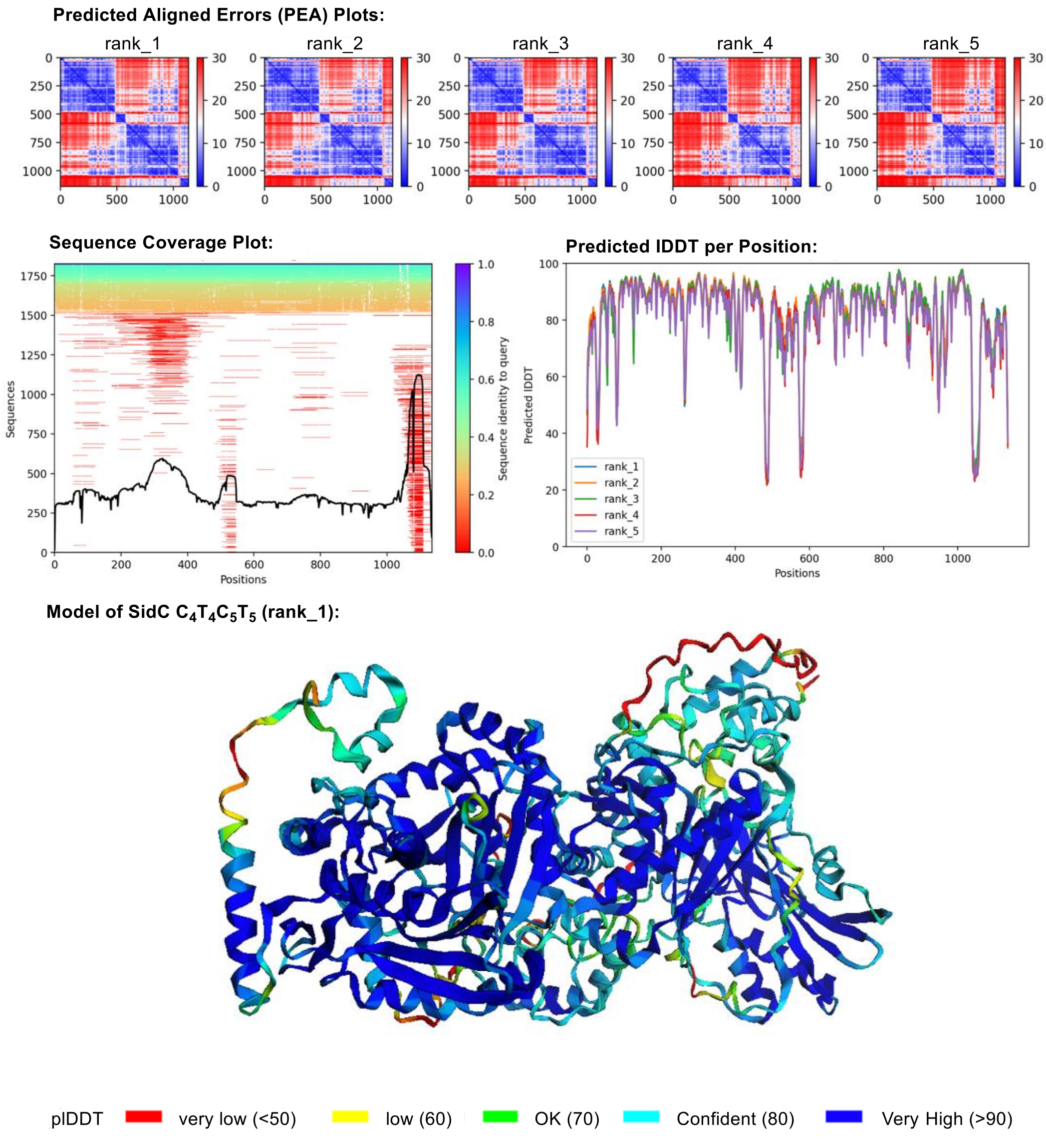

Supplementary Figure 15. Structural assessment of AlphaFold model for SidC $C_4T_4C_5T_5$ fragment. Predicted aligned errors (PEA) plots for the top five ranked models are shown, in addition to sequence coverage and predicted local distance difference test (plDDT) plots. pLDDT values are visualized on the rank_1 structure of SidC $C_4T_4C_5T_5$. The core catalytic domains have pLDDT scores >80, whereas linker regions between domains have scores <50 consistent with previous observations of their disordered nature.[1] A co-ordinate file for the SidC $C_4T_4C_5T_5$ region is available for download from Mendeley Data DOI: 10.17632/c3ymyp3yx4.1.

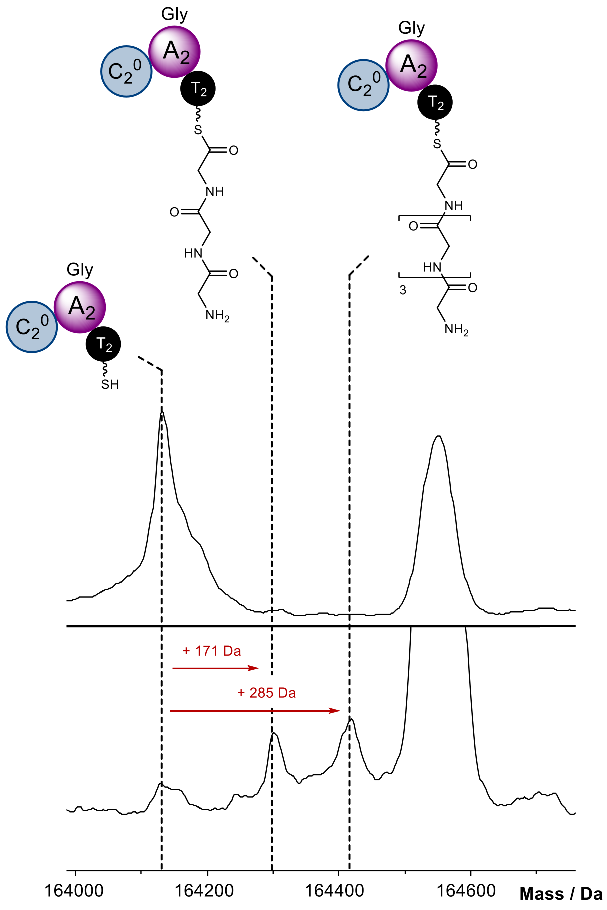

Supplementary Figure 16. SidC $C_2{}^0A_2T_2$ retains ability to produce Gly_3 and Gly_5 thioester intermediates. Deconvoluted intact protein mass spectra of *holo*-SidC $C_2{}^0A_2T_2$ (*top*) and *holo*-SidC $C_2{}^0A_2T_2$ (*bottom*) following incubation with Gly, ATP and Mg^{2+}. The spectrum shows a set of peaks corresponding to Gly_3 and Gly_5 intermediates in an identical fashion to Fig. 3b, spectrum iii. Note – the SidC $C_2{}^0A_2T_2$ mutant gave reduced yields of protein, and therefore lower signal intensity in the MS. Mass shifts corresponding to biosynthetic steps are highlighted with red arrows, and proposed intermediates are displayed. Exact measured and observed masses are detailed in Table S3. Experiments were performed in duplicate and representative data are shown.

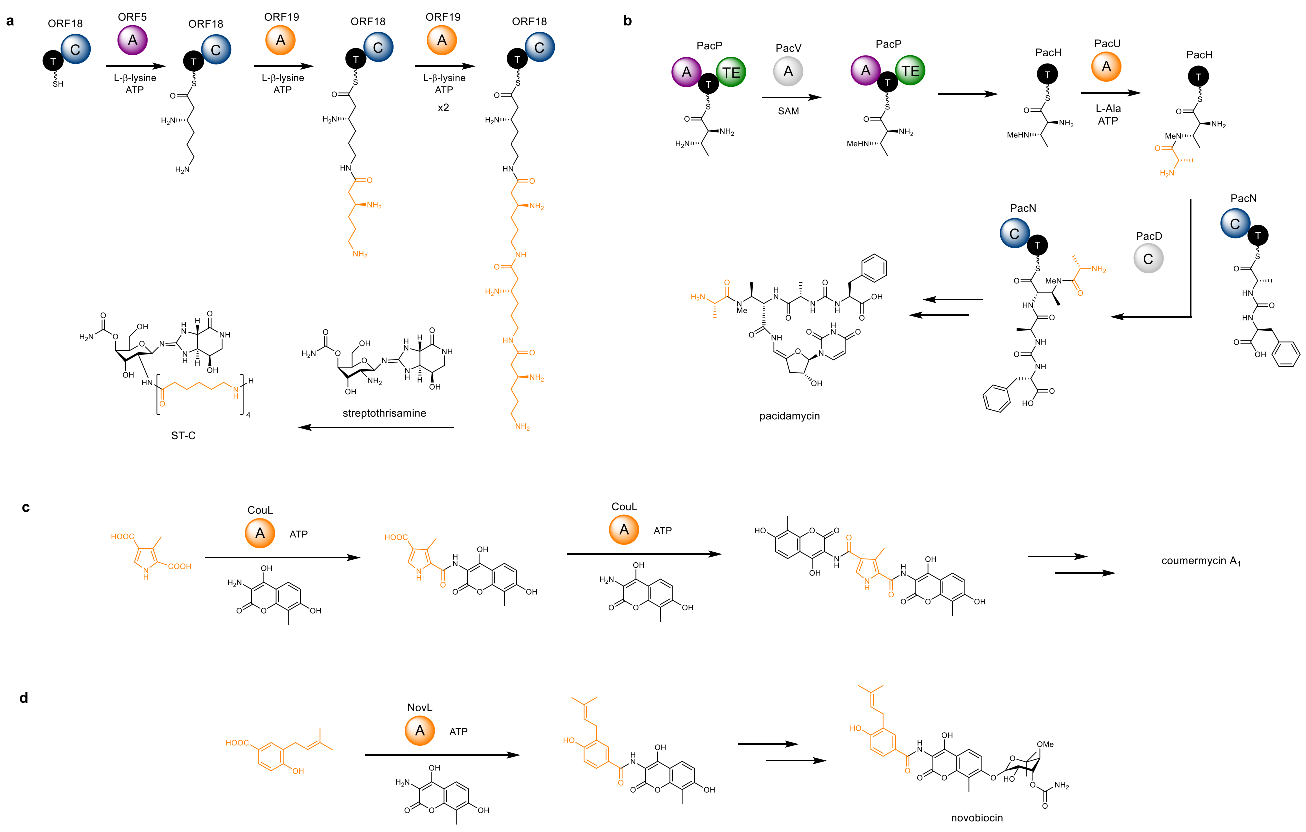

Supplementary Figure 17: Examples of other amide bond-forming adenylation domains in natural product biosynthesis. Partial biosynthetic schemes highlighting key amide bond-forming steps conducted by adenylation domains for a). ST-C, b). pacidamycin, c). coumermycin A_1 and d). novobiocin. In all cases, the amide bond-forming adenylation domain and extender unit(s) are highlighted in orange.

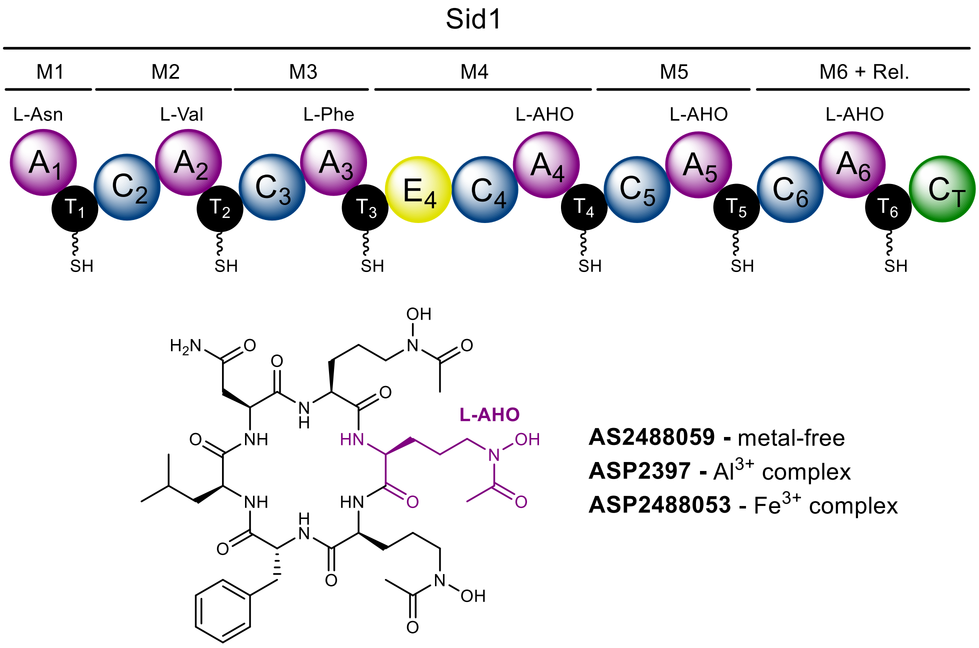

Supplementary Figure 18. Domain organization of the Sid1 NRPS responsible for biosynthesis of AS2488059. Unlike most members of the ferrichrome family of NRPSs, Sid1 presents a colinear relationship between domains and product. In each case the predicted specificities of the A domains are shown (based upon a linear biosynthetic model). It is also worth noting that Sid1 harbors an epimerization (E) domain, which inverts the stereochemistry of the T_3-bound L-Phe to D-Phe.

Supplementary Tables

Supplementary Table 1. Primers used for the cloning of SidC constructs and mutagenic primers to generate point-mutations/truncations.

Primer	Sequence (5'-3')
pLH-SidC-WT-F1	TGGCTAGCCATCACCATCACCATCACCATCACACTGCAATGGGGAAACGTAAGCTGGCTG
SidC-WT-R1	GATATTCCCTCCAGAATTTGGATGTGGAAGTGTG
SidC-WT-F2	CACATCGTCAAACACCACTTCCAGGCAG
SidC-WT-R2	CTGCGACTTTCTCTAAAACTGGGAAGGACG
SidC-WT-F3	GTCAATAACCTCGTCCTTCCCAGTTTTAGAGAAAGTCG
SidC-WT-R3	CCGCAATAATGAGTCTCGAGATCTGTCCTTG
SidC-WT-F4	GAAATGAAAAACAAGGACAGATCTCGAGACTCATTATTGCGG
pLH-SidC-WT-R4	AATTAGTGATGGTGATGGTGATGCACGTGAGCTTTTAAGCAAGCAACAGCTCTTTCAATC
pLH-SidC-T_1-F	GCTAGCCATCACCATCACCATCACCATCACAGTGAAGAAAGTGCAGAATGTTGGAGGCAT
pLH-SidC-C_2-F	GCTAGCCATCACCATCACCATCACCATCACAGTATAACAAACGACTTGCAGTTAAGACTG
pLH-SidC-A_2-F	gCTAGCCATCACCATCACCATCACCATCACGAAGTCAGCGAAGGAGCACTCC
pLH-SidC-C_3-F	TAGCCATCACCATCACCATCACCATCACGATCTTGTCAGATTAGCCCAGTCTACTTC
pLH-SidC-T_4-R	CGCGCTGCAAACTAATGGTATTTAATTTAAATGACAAATTTGTGCGGACCGATGCC
pET-SidC-A_1-F	TACTTCCAATCCAATGCAGCAATGGGGAAACGTAAGCTGGCTG
pET-SidC-A_1-R	TTATCCACTTCCAATGTTATTAACTTTCTTCACTGAGGCCATTCTTGATTGG
pET-SidC-T_1-F	TACTTCCAATCCAATGCAAGTGAAGAAAGTGCAGAATGTTGGAGGC
pET-SidC-T_1-R	TTATCCACTTCCAATGTTATTACTCGCCATTTTGATTCGTCAGCATGCTC
pET-SidC-C_2-F	TACTTCCAATCCAATGCAAGTATAACAAACGACTTGCAGTTAAGACTGCAGTC
pET-SidC-A_2-F	TACTTCCAATCCAATGCAAGGGAGCACTCCTACAATCACAGTTCG
pET-SidC-A_2-R	TTATCCACTTCCAATGTTATTACTCAGGGTTAACGTTGTTCTCCCACTTGTCAATATC
pET-SidC-T_2-F	TACTTCCAATCCAATGCAGGAGATGCAGATGAGGATGATGCTGCC
pET-SidC-T_2-R	TTATCCACTTCCAATGTTATTAACTGAATGAAGTAGACTGGGCTAATCTGACAAGATC
pET-SidC-C_3-F	TACTTCCAATCCAATGCAGATCTTGTCAGATTAGCCCAGTCTACTTCATTC

pET-SidC-T_3-R	TTATCCACTTCCAATGTTATTATCCATGCTCAGAAGCTCTAGGCAACC
pET-SidC-C_4-F	TACTTCCAATCCAATGCAGGAGTTAATGATCATGCAGATTATTTTGCACAGCG
pET-SidC-T_4-F	TACTTCCAATCCAATGCATCTGCGTCTCATGGTGCATCCGTACAATG
pET-SidC-T_4-R	TTATCCACTTCCAATGTTATTACTCCGTTTTCCCAATACCATTAGTTTGCAGCG
pET-SidC-C_5-F	TACTTCCAATCCAATGCAACGGCTATCTCAATCGAAACCTATGCAAA
pET-SidC-T_5-F	TACTTCCAATCCAATGCAGATTCTGCGCCCTCTACGCCTGTG
pET-SidC-T_5-R	CCGTTATCCACTTCCAATGTTATTAGGTAGAGACTTCGGATTCTAAC
pET-SidC-C_T-R	CCGTTATCCACTTCCAATGTTATTttaAGCTTTTAAGCAAGCAACAGCTCTT
pET-SidC-C_2^0-F	CAGCATGTTCGCTGGAATCTTCGACG
pET-SidC-C_2^0-R	CAGCATGTTCGCTGGAATCTTCGACG

Supplementary Table 2. Measured and calculated (in brackets) masses of *apo*-, *holo*- and haOrn-bound species detected by intact protein MS. All calculated values are average protein masses.

Protein	*apo*-	*holo*-	*holo*-L-haOrn	*holo*-(2 x L-haOrn)	*holo*-(Gly_3-L-haOrn)
**SidC $C_3A_3T_3$**	126,449 Da (126,452 Da)	126,790 Da (126,792 Da)	126,963 Da (126,964 Da)	-	127,136 Da (127,133 Da)
**SidC $C_3A_3T_3C_4T_4$**	188,652 Da (188,656 Da)	189,331 Da (189,336 Da)	189,506 Da (189,508 Da)	189,680 Da (189,682 Da)	-
**SidC $C_3A_3T_3^0C_4T_4$**	187,571 Da (187,574 Da)	187,911 Da (187,913 Da)	188,085 Da (188,086 Da)	-	-
**SidC C_4T_4**	60,270 Da (60,271 Da)	60,611 Da (60,611 Da)	60,785 Da (60,784 Da)	-	-
**SidC T_4**	9,911 Da (9,912 Da)	10,251 Da (10,252 Da)	-	-	-
**SidC $C_5T_5C_T$**	117,487 Da (117,492 Da)	117,827 Da (117,832 Da)	118,000 Da (118,004 Da)	-	-
**SidC T_5C_T**	64,641 Da (64,644 Da)	64,980 Da (64,984 Da)	-	-	-
**SidC $C_2A_2T_2$**	163,865 Da (163,866 Da)	164,205 Da (164,206 Da)	-	-	-
**SidC $C_2^0A_2T_2$**	163,799 Da (163,800 Da)	164, 138 Da (164,140 Da)	-	-	-

Supplementary Table 3. Measured and calculated (in brackets) masses of Gly-bound species detected by intact protein MS. All calculated values are average protein masses.

Protein	*holo*-Gly	*holo*-Gly_2	*holo*-Gly_3	*holo*-Gly_4	*holo*-Gly_5
**SidC $C_2A_2T_2$**	164,262 Da (164,262 Da)	-	164,378 Da (164,377 Da)	-	164,490 Da (164,491 Da)
**SidC $C_2^0A_2T_2$**	-	-	164,307 Da (164,311 Da)	-	164,421 Da (164,425 Da)

Supplementary References

1. Farag, S. *et al.* Inter-Modular Linkers play a crucial role in governing the biosynthesis of non-ribosomal peptides. *Bioinformatics* **35**, 3584–3591 (2019).
