# Supplementary material for: Elucidating the molecular programming of a nonlinear non-ribosomal peptide synthetase responsible for fungal siderophore biosynthesis
Source: Nat Commun. 2023 May 17;14:2832. doi: 10.1038/s41467-023-38484-8 (PMC10192304; doi:10.1038/s41467-023-38484-8)
Supplement: Supplementary file 3 — Description of Additional Supplementary Files [file 41467_2023_38484_MOESM3_ESM.pdf]

File Name: Supplementary Data 1
Description: DNA and protein sequence for SidC construct used in this study.
