# Supplementary material for: Elucidating the molecular programming of a nonlinear non-ribosomal peptide synthetase responsible for fungal siderophore biosynthesis
Source: Nat Commun. 2023 May 17;14:2832. doi: 10.1038/s41467-023-38484-8 (PMC10192304; doi:10.1038/s41467-023-38484-8)
Supplement: Supplementary file 4 — Supplementary Data 1 [file 41467_2023_38484_MOESM4_ESM.pdf]

**Supplementary Note**

Numbering of amino acids excludes the His-Tag regions (highlighted in grey), and correlates to numbering referenced in the main text.

## *sidC* NRPS (DNA)

ATGGCTAGCCATCACCATCACCATCACCATCACACTGCAATGGGGAAACGTAAGCTGGCTGGCTCGTTCCGCAGATATGTCGCTACCGGGCTTTCTCGGGTCTCGGGCGCTTTTCGG
AGTCGCAGTGCCAGTAAGGAACGCCAGAGCCGTGGCAGCGGCAGCTTGGTCGAACGTGATACGCTGGCAAAAGACCAGGGCCAGGGGGTCTGTTTACAGCCAGCTCAAGTTGGAG
CTGTCCCTGTCACTAAGGTCCCGGCGCTTGATACGATCGAAGCATATGCCGGAGCATTTGATAAACCAATTGACGGGGCCGGTACGTGCCAGCGGACAAGTTTGGTCTGCAGTGCC
AGCTCTGCCGTTGACGGTGACGTGATTGACGGCTTGGTTCAGGGCTATGCGCGGTTCATCGCTGGATTGACGGGTCTTGACGATATCGCGTTCTACTCCACTCGTCATGAACCGTTT
GCATTGGAGAAGTCGACATCAATCATTATCGCTTCTGCTGCTTCCAACGAGTTAGTCTGTCGTGAGGTGGACACGAACCACGAGAACGATGTGCAGTTCTATGCTGAGCTGGGACGG
GTCGAGCCTGAGAATGGACGACGTGAATTCCAGCCGAATGCATTCACACTCTTCATCGAGCCGGACAGCAATAGGAAGAAGAAGAATGTTCTTCACCTTAGCTTCGCCTATCCTCGAC
GGCTGATCCCAGATGCAGCGGTAGAACAGCTACTGCGCACGCTTTTACTGCACATCTGCGAGTCTTCTCCACTAAAGTCGCCATCGACATCGCAGCCTGAATTGTCCATTCTGAATTT
CCCGCCGTCTATGATACCCCCAACTGCGCAGGCGAACGGCGTCGAGAATTCGACGACCAACCCGCATCTCTTGCATTCCGCATTCGAGAACTGGGCTCGCAAGAACCCGCATTTTAT
CGCATTAGATTTCATTCATTCCCTTTCATCCAAAACGCACAGATCAGAGCATAGCATCATCACTTATGCGGCCCTCGATAGCGCTGCTACCAATCTAGCCTTCCATATTAGATCACTTCT
ACGCGACTCTCGAAAACATGGACAGATTATCCCTGTTCACATGCCCACTTCACCGGAGCTCTACATCTCATACCTCGCCGTCCTGAAAGCTGGCCACGCTTTCTGTCCTATACCTCAA
GACGTTCCTGCCCGACGAATACAGGAGATTCTTAGCGATATTGATGCTCCTATTGTCCTCGGGACCTCATCAAAACCGCCCATCAGTGCTGAAAGCTCCCGGTCGACGTCTACATGG
GTTAATGTCACCGAAGTTTCCAAATGGCGGCAGATGTGTGGCGAGCAACCAGCTGATTATTCTCGACCCTCTTTAGACCACATAACCATTGAGCAGAACCAGACTGCTTATCTCCTCTT
CACCAGCGGTTCAACCGGTAAGCCGAAAGGTGTGCAAATCAGCCATCTCGCAGCATCATGCTCCATATCCTCCCACGCCACTGCAATTCCTCTCCCCGGCGAATCACCAGGCAACTT
CCGCTGGTTCCAGTTTGCTTCGCCATCCTTCGACCCTTCACTCATGGAGATTTTTGTCACCCTAAGCACTGGTGGCACGCTCTGCTCTGCTGATCGCAGACTTACCCTCGCCAATCTC
GAAGCCACCATCAATGAGAGCAGAGCGACAGTCATGATGGCTACGCCGAGTCTTGCCACGCTTCTCAGACCTGACCGGCTGGAGACCCTTGAGGCGCTGTGGTCCATGGGAGAAAA
GCTCAACCGGACAGTTATTGACAACTTCGCGCTGGATAATGTTATGAATGGAGACGCCGAGACCAGAAGACCGAGGACCTTGGTCAATGCATACGGTCCAACGGAAGGAGCAATCAA
CTGTACCTACGTCGCTCCCTTCAAGCGTCACATGCGTGGTTCTATCATTGGACGGCCGCTACCAACCTGTGCGATGTTTATTCTATCCCCCGACTCCCAAGTCCCTGTCTTAGTCCCT
ACCGGTACAGTTGGAGAACTGGCAATTGGCGGCCCCCAAGTCAGCAAAGGCTATCTCAATCTCCCCGAAGTAACAGCTAGAGTCTTCATTCGCAGCAAGGAATTTGGACCCCTCTAC
CGCACTGGCGACAAGGCCCGCATCGTCTGGGACGAAAGCGGGCACCAGGTCATTGAGTACCTAGGCCGAATCCGCACGGATCAGGTCAAGATAAATGGACGCAGAGTTGAGCTGG
GTGAAATTGAGTCTGTTGTCGCGGCTGTGGAGGGTGTTCGGGAGGCCGTTGCGGTTGTGGTAAAGAGAGATAGCAAGTCAAACGGGGGCGAGCAGATTGTCGCTTGTCTTGTGGTA
GATGCTGCGGATGGGGAGGGGCGTGAGAAAATTGCACAGCAAGCAAAAAAAAATGCTGCGCAGCATCTTGCGAGCTTCATGTGCCCGACTACCTACACATTTTTTGATGTGCTGCCC
AGGTCGAGTTCCGGAAAGGTCGATCGAAAAGCGTTGGCTGTCCAGCTGCAAGAGAAGCCTGATGTTCCAATCAAGAATGGCCTCAGTGAAGAAAGTGCAGAATGTTGGAGGCATTCA
GATGAGGCCGCATCGTCAGTACAGCAGCTGGTGATTCGACTCGTTGCGGAAACAGGGGACGTGAGTGATTCCGCAATAAAGCCCGGGACAGAGCTGTACTCGGTAGGCATCGACAG
TCTAGGAGCAATGCGTTTTCTACAGAAGCTGAGAGACAATGGGGTCCATGGGCTGTCCGTCGGCGAAGTGCTGCAAACACATACATGCCAGCGGCTTGTCTCTCTCGTCCAGAGCAT
GCTGACGAATCAAAATGGCGAGCCTAATGGGATACTTCAGAAGGGCAGTATAACAAACGACTTGCAGTTAAGACTGCAGTCATTCGGCCGGCGATATCGCATGTTTTGCGCAGAATC
CCTTGACGTGCCCGCGGACACGATACATGAAGTCCTTCCTACCACTGCTACTCAATCCGGGATGTTGACTAGTTTTCTGCGTAGCTCTGCGGAACAGTCCTATGAGAAACCAACATAC
ATTTATCATACTGTCCTTCCACTTGAGCCCCGTACCAACATTGAGAAGCTCAAGAAGGCCTGGTTCGATGTTATCTCAAACTATGACTCTTTCCGGACGGTCTTCTGTATGGTCGACGA
TGAGCTGGCCCCGTTTGCACAGTGCATCTTGACGGCTGAGGGCGTCTCATCCAGGGACTGGAACGTTTACACAAGCCCTAACGAGTGCTCCGCAGAAAACGACATTATCGACCACG
CTCTTCGGAGTGCTGAAGAATCAATCACATTGAGGAGACCTCCCTGGAAGCTGTCTTTGGTGCAGTCATCGACGAAGTCCATCATGATCCTCAGCATGTTCCACGGAATCTTCGACGG
CGGCTCTCTACAGCTCCTCTTGCAAGATGTCTCGTCAGCGTACTCCGGAAACACACTACCGCAGCGAACCTCACTAACCCACATCGTCAAACACCACTTCCAGGCAGATCACACTTCC
ACATCCAAATTCTGGAGGGAATATCTACAAGGTTACTCTCTCCTCCCCTTTCCATCTCTCACTCCGCATCGAGCGCCGGCCCAGAAAAGCACTGGCTGTGCTGAGGTCACCTCTCAAC
TCAGTTACGGAGCCCTCAAAAAACTCTCAAAAAGCATTGGCTCTACACCACTCTCTGTGCTGCAGGCAGCATGGGGTGCGGTAGTTCTCAGCTACACAGCTACGCCGGATCAGGACG
TAGTTTTGGGAAGTGTGATGTCTGGACGACTTGATCCCGATTCGAAAGACTGCATTGGTCCGACCTTTACGATAACGCCTTCCAGAATTTCCGTCCAGCAATTGAAGGGTTCCGGATC
CTTGACAAACCAATCAGTGGTCCACTATCTGTCGTCTTCAAATGCAAAAGCGCTCTCACACCTCCAGCCTCAGCTAGGATCTGTGACCGTAAATGGCAAGGTTCCCTATGACACAGTC
CTCGCATACCAGGACTTTGACACTGATATGAAACCTTCTCAAACCTGGAGTTCTGTCCAACATCCCGCTATGGCAAATGATTTCTCTGTGATGATCGAGGTGGTCCCCAATCCGGACT
CGACGTTGACGCTGCGGGCTAGTTTCGACACGAAACTCGACTCTACCGGAGCTCAAATCATGCTGAAACAAATGGATGATATCGTCAGCTACATCCTCAACCACCCGGACTCGAGTTT
CGAAGATGCTCCTCTTCAAGCCAGCTTAGCTCTGAAATCGAAAGCGAACCCCAGTCCAATTACAGCTCCAGAAGTCAGCGAAGGAGCACTCCTACAATCACAGTTCGAAGACCATGC
CCTCTCGCATCCAAATGACCCAGCTCTAGTATTTAAGCAGGACCTTGATAATGATGATCATCCAGGAAACATCACTTGGACATATGCCCAACTCAACGCTATGGCTGAAGCTCTTGCG
GAACATCTGCTCCAGGTTTGTGGTGATCTGAGAGATGCTTCTGTTCCGATTTGCATCGAGAAGAGTCCACCCTTGTATGTTGCGATCCTAGGTATCCTCAAGGCAGGAGGCGCCTGGT
GTCCCATTGATACACTTTCCCCAGCACAGCGTCGCCATGATCTCATTGCGCGAACTAGTGCCGGTATCCTTCTGGTTTCTGGTCTCGATACGCCACAACCTCAGAATGCTGTTCCAGC
TGGAGTCAGAGTTATTGATGTTAGCAAGTTCATTCAGAATGTCTCGAGCGACAATACCCCACAGTCAAGCAGACACCGAGCAACCCCTAGAAATACAGCCTACCTAATCTGGACGAGT
GGCACAACTGGCGCTCCCAAGGGCGTACCCATTACGCACTCAGCCGCCGTCTCTAGCATGCGCTCCCTCCAGACCGATATCCCGGGTAACGAAGACGGCAGTCCCATTCGCTGCCT
CCAATTCTCGCAACCAACCTTCGATGTCTCCATCCAAGACCTCTTCTACACTTGGGGCTTAGGAGGCGCGCTTATTTCTGGCACGAGGGAGATAATGCTCGAATCTTTCCCTAAACTT
GCAAATATCACCAAGGCAACGCACGCCCATCTCACGCCTGCCTTTGCGGCTGGTGTCGCGAGGAAATCATGCAAAACGCTTAAAGTCGTAACAATGATCGGCGAAAAATTGACACAG
AGTGTTGCGGATGATTGGGGGACAGACATGAGGGCGTTCAATACCTACGGCCCCGCGGAGGCGACGGTCGTCAGTACGATCCGCGAATTCGGCAATGAACATAGATCCGTCAAAAG
TGCGAATATCGGGTGGCCAATGAGCAGTGTTTCGGTCTTCGTGATGTCCAAAGATAAGCGCGTGCTCATGAAGAATGCCATTGGAGAGCTTGCATTAGGTGGCCCTCAGCTTTCACC
AGAATACTTGAATTTAAAGGACGTAACGGACACGAAGTATATCTGGAATGAGGACGCTGGGCAGCGGCTTTACTATACGGGTGACCTTGTCCGCATGCTATCTGATGGGAGTCTGGA
GTACATCACGCGTGTTGATGACCTTGTGAAACTAGGTGGCATTAGGATTGAGTTGTCGGAGATAAGTTTTGCGCTGAGAGGGTGTCATGAACTCGTCGAGAGCGTGGAGACTATGAT
ACTTAGCAGGAAAGACAGGCCAGTCAGAGTTGTTGTTGCGTTCTTGTGCGCCCCGAAGGCTGCTGGTGATGCGGATGAAGGTTTGTTGGTGCTTGACGACACTGGTAGAGATATTGC
CCGTGCAGCAAGCCTTCAGGCGCGCAATGTCCTGCCAGAGAACATGATTCCCTCTGTGTATCTTATTGTCAAGAAGATACCAAAAACGCCATCTGCAAAGGTCGATCGACGCGCTTTA
CAGGCTGCGTACGCAGCGGTTGATATTGACAAGTGGGAGAACAACGTTAACCCTGAGGGCCCCGGAGATGCAGATGAGGATGATGCTGCCACGGCCACTCAGATCATCGAGACAGT
GGCAGCGCTGGTGCATGTTGAGAGCTCGACTATCACAAAGAGCAATAGATTACGCAGTCTAGGTGTTGACTCACTCTGTGCCACGCGTCTTGCATTTAGGCTCAAGGAGGCCGGGTT
TGGTCTTTCTGTTATGGATGTCCTGGCCTGCACCACTATCCAGGATCTTGTCAGATTAGCCCAGTCTACTTCATTCAGTTCATCTGCAAACTCAGCTAGCCCTCACAACGACAAGTTCG
ATATCGTCTCCTTCAACAAGATATGGCATACCCTCGTCGCAGCAGCAGCAAAGATCCCTGAGAAGGACGCGTTTACAACGATCCGCGCCACAGCTATCCAGGAGAGCTTGCTTACTG
AGACAATGGGTACATACGACATGTACTGGAGCAATCATTTCTTCAGACTTGATAGGTCTGTCGACATTCCCCGACTACGACAAGCTTGGTATGCCGTGTGCCAGAAGACTGAGACGCT
CCGGACGGGGTTTATACCTGTCGCACAGACGGAGGCAAAGAATAAGAAACAGGCTAAGGATTCGGGATTTTCTATTTTGCAGGTTGTGTATAAGCTTCCGGCGGTGGATTGGGAGGC
TCATACCTACAGGAAGGAAGAGTGGAGTTGGGTGCTAAAGAACCGAGTGCGGGATATCATGACGGCGCACCAGAAGAACTACTTCTGCCATCCTCCTTGGGCAGTCACAATACTGGA
AGAAGGCACCGAACGAGTCATGGTCTTGACACTCCACCACTCAATCCATGACGAGCCTTCCCTCAAGTTCTTGATGGACGATGTCCGCGCCGCATATACCTACAAACCGCCTCTTCG
CACCCAGCTGACCCCCGCCCTCGCGCTTGTGCTCCCAACTCCAAGCAAATACGCCGAAGCTGTTGATTTCTGGTCCTTGGAGCTCAAGCCGTACGCCGCCCTCGACGTCCCCGTCT
GGCCCGATCTCACAGGTAAACGGGTCTCACCAGGCGCGGCGCCAGAATACAAACTGATATCCGAGGCGATGTCAATAACCTCGTCCTTCCCAGTTTTAGAGAAAGTCGCAGCAGATC
TAGGGCTGAAATCTGTTGCGTCGATCATTAGGGCGGCATGGGCGTTCGTCAGCCTGTCTTATCTGGGCCTGTCGGGGACGGTGTTTGCGGAGACGTTATCAGACCGCGTCTTTGATC
CGTCCTTGGAAAGCGCAGTCGGTCCTTTTATATCTGTTGTTCCGGTTCCGTTCCGAGTAGAAGGGGATACGACAGTGAGGAAGATCCTGGCAGAACAGCACCGGCTCTCCTTGCAAT
CGTGGAAGCATCGACATGTTCATGCGAGAGATATTCGGAAGGCCCTTAAGCGGCAGAGAGGAGAGCCGTTGTACCCTGCAGTATTCAATTTCCACGCGCTTGACGAATCAAAGGACA
GAAAAATTGCGAGCCTGCCTGGTCTCTGGCATGAGCTTGAAGACCAGATTGGCCTGCATGTTGAGCATCCCATGGCCATGAATGTTTTCCAGTCGCCAAGCGGAAACATGACCCTGG
AGGCATCAAGCGATAGTCGGATCTTCAGCCGTGAACATTTACGCCTTTTTGTTCGCCAGATCGATGCTCTCATCTCGGAAATGCTCTTATCGCCTGATGAATCTCTCAGCGGGCTCGT
GAATCGGCTTCCGTCTGACCTACGATCCCTATCCAACAGGATTGTGAGCCATGAAGTCGCGAATTCAATTCACCAGGCCCCAACTTACTGGCTGGAGAAGTTCGCCGACACTCACCC
GCACTGGACAGCCGTCGAGGTGGCGTCCAGTATCAGTACAAACGGAATTGAAAAAGAAGCCATGAGTTATGGCTCTCTAAACAGCGCAGCAAACCGTGTTGCTGCGTACTTGGCTTC
ATTTAGGTACAAGAATCGAGTCGTCGGTGTCTGTGCAGGACGGACATTAGCGTCGTACCCCATCATCATTGGCATATTCAAGTCCGGGAATACATACCTTCCTATTGATGAGAGTCTG
CCAGCCGATCGAAAGGCGTTCTTGCTGGAGGATGCAAAGTGTCCGGTTGTCTTTACGGAACTGGGTCTCAGGAATTCCTTTGCCGGTGCTCCTGATACCTGTCGCGTGGAGTGTATT
GACGACCCTGCCCTGCAGAGATCATTGGACGAAATGCCATCGACAAACAAAGACTACAGCTCCCATCCAGATGATGTCTCATACCTGCTATTCACTTCCGGCTCGACCGGCAAACCC
AAAGGCGTCATGGTCACTCGCGCGAACCTGTCGTCCTTCATCGAGTCCATATCTGAGTTTGCATGCCGCATTGCACCAGATACGCTTAAGCTGGGTGGCACAGGTCGCTATCTAGCG
CAAGCAAACCGCGCCTTCGACCCGCACCTGCTGGAGATGTTCTTCCCATGGCGCCATGGCATGGCCACTGTGACTGCCCCGCGGCCTATGATCCTTGATGATATCGGGACGACTCT
ATCCAAATGGAGCATTACACACGCCAGTTTCGTTCCCAGTCTCGTCGACCAGAGCGACATCACGCCCCAGCAATGCCCCAACCTGCGGTTCATGACAGTGGGCGGGGAGAAAATCA
CACAGAAAGTCCTCGACACCTGGGCCTCAGCACCGAACGTGGCTATCGTGAACGCCTATGGTCCCACGGAGGTAACGATTGGCTGTACATTTGCGCATATCAACCCGTCCACAAACC
TACGCAATATCGGCCCCCCTCTTACAGCTTGCACAGCGCATGTTCTGATCCCTGGGACCATGAAGTACGCACTTCGGGGCCAGACTGGCGAATTATGTTTCAGTGGTGATCTTGTGG
CGAGAGGATATCTGAATCGCCCTGATGCCACTGCCGCAAATTTTATCACTGGCCCCAACGGTGCCAAGATGTACCGTACCGGTGATATCGGCCGGCTTATGTCTGATGATTCGGTTG
AGTACCTTGGACGTGGAGATGACCAAACCAAGATCCGTGGGCAGAGACTAGAACTCGGCGAGGTTTCCGAAGTACTAAGAGCTTCTTCGCCAGTTGCCGTTGATATTGTGACGACGG
TAGCGAAACATCCTGACCTCGGCAAGGTACAGCTGATTACCTTCGTGTCGAGAGCAAAGAAGCGCACTGTCAATGAGGAGGTGCAGTTTCTATTCTCTGATTTCGGTACCCTAGGCCA
AGAACTGCGCGATATTTGTGCGAAAAAGCTGCCAGCCTATATGGTCCCAGACCTCATCTTGCCCGTGACAAGTATCCCCGTCGCGGCAATGAGCGGAAAGGCGGATATGAAGGTATT
GCAAAAATTATTTACCGAGCTGCCGTTGCAGGTTGTTCTTCAGGGGAATAATGCTATTGCAAACGGAACTGGCTCCGAACGGCCGCTGAACCCAGACGAGATAGCCGTCGTCGGCGA
GATTTGCCAAGTTATCTCTGCTGACTCACACAGCTTTTCGCCTATGACGAACATCTTTGAAATTGGGATTGACAGTCTCAGTGCAATTGGGCTCTCGGTTAGACTACGTGGAATTGGAT
ATGCGGCGAGTGTGGCGGCAATTATGGCCAACCCGGTTGTCGAGCAGCTTGCTAGGTTGCCTAGAGCTTCTGAGCATGGAGTTAATGATCATGCAGATTATTTTGCACAGCGGTGCA
AGGAGTTGGAAAGCCAGTATCGAAGCGTTTTTAGGGATGCTGTCGAGGTTGCCGTAGTCAGGCCTTGTCTCCCATTGCAGGAAGGACTGATTGCGCGCTCTATGAACAGTAACAGCG
GCGATGGCAAGCTCTATGTCAATCATGTCATTCTGCAGCTGAACAAGGATGTTGACACCAGAAAGCTAAAAAGCTCTTGGGAGGATGTGGCGAAGGAGAACGAGATATTGAGGACGG
CGTTTGCGCCACTAGAGAAGGAGATTGCGCAGGTTGTCCTGTCCAATGCTTCTTATCAAATGCAATGGACAGAGGGGAAGTATGAAGATTTGGATGAAGCCATTCAAGCACGAAATGA
AAAACAAGGACAGATCTCGAGACTCATTATTGCGGATTTATCAACTGTTCCTCCGGCACGGTTCCATCTTGCCAGCTCGGCGAGTGGGAAGCCGCTGGCTTTGTTTATTTCGATACAT
CACGGGCTCTATGATGGCGAATCCTTTGCGATGATGCTGGACGAGGTTGCGGCACGGTATGAAGGGAGGAAGGTCGGGGAGAGAGGCTCACCGTCTGTCTTTTTGAGGCATGTTTG
TTCCCAGGACACAGAGGAGGCGAAGAGGCACTGGATGCAGCAACTTTCTGGATGCACCCCTACAATATTCCGAGCCAACGGAAATGCAATCAAAAACACCATTTCCATTCGTCGAAC
TGGCAACGCCAAACTCTCGGACTTAGAAACCCGCTCGTCAACACTCCAGACGACAGTCCCGAACCTGCTGCAGGCAGTCTTTGCTCTCTTGTTAGCGGACCACACCGGTATTTTCGA

TGTAACCTATGGCCTTGTCCTGTCAGGACGCACTATATCCGCCCCAGGGGCGGACTCGGTACTTTTACCGGCTATCACCACTATCCCTGGGCGTCTCAACATGAGCGACTTGAAAAC
CGTTAATGACGTGGTGAAGGTTGTTCAGAGAGCTACCGCGAGATCTTTAGACTTCCAGCATACACCCCTCCGCAAGATCCAGCAGTGGCTGAAGTCCGAAGCCCCATTGTTCGATTG
TCTGTTCTCGTATATCCGCGCCACTGCTCCACCTGGCCATAACCTCTGGGCAGAATTGGACAGCCACATGCCGTCTGAGTACCCACTTGCCCTTGAAGTGCAAGCCGATAATGCGGC
TAATACACTCAAATTGGAGTGCATCTTCTCCTCAGACTTTGGACCACGGCAAGTTGGTGAGGAATTCCTTGAGAAGATGGACGCTGTTATTTCTGAAGTTGTCTCTGGTTCAAGTTTGC
CTTTGGAGAACTTCAACACCGTGCGATCAGCTACCTCTGCGTCTCATGGTGCATCCGTACAATGGGACGAGTCAAGCTGGACTTCGTCAGAAAGCAGGATACGAGAAATAACGGCAA
CGTTCTGTGGACTGAATGTCGAGGCTGTATCAAAAGGCGCTTCATTCTTTAGTCTTGGCATAGACTCAGTCACTGCTCTTCAGTTTGCCAGAAGGCTGCGTGATGAGGGATTCAAGGT
TTCATCATCGGAAATTATGCGTTTTTCCTGTGTTGGGTCGTTGACAGGACATATTGAATCTTCTTCCGCGCTGCAAACTAATGGTATTGGGAAAACGGAGACGGCTATCTCAATCGAAA
CCTATGCAAAGCATATTCCTCTGCTTGGAAAGAATGACTCCATCACGTCGTTGTTCGAATGTACACCACTGCAATCAGGAATGATCACGCAAACGATCAGCTCTGGAGGGAAGGTGTA
TATTAATCCTCATCCAATCCGTCTCAGGGACAACGTGAAAGTGGAGAAGCTCAAGGAGGCCCTGCGTCACGTTGTTCAGGCTAACGAAATTCTGCGCACGTCTTTTCACCTCATCCCG
GATCTAGGAGAGAGCTGGATTGGTGCCGTGCATGAAGAGCCTAAGTTTGAGTGGAGTGAGATCAATATGCCATCAGGCGCCAATGCTCTGTCTGAGGTTATGAACCTCTATACGTTCT
GTGAAGAGGCATCTTTTGAGAGGCCACCGATTCGTTCTGGTCTTGTCAACCAGCCAGGCTATAGGATCCTCATAGCTGTCCTACATCATTCCCTTTACGACGGTGCCTACCTACCGTT
CGTCTTCGAGGACCTTGCAACAATCTATGCAGGAGGAACCCTTGCTCAACGACCACAGTTCTCAGAAATGGTCCCCTACATGCTCTCAGGGCAAGACGAATCCTGTGCCTTCTGGGT
GGACAGGCTCAGAGACTATGTACCTGTTGAGATCTGTCCACTACCCAAGCTCGACAGCACTCCAAATATGCTAACCGCGAAAAATCGCATTCCCCTACCCCTCCACTCAATCACAGAA
TCCTGCAAGACCATGTCCGTCACAATCCAAACTGTCTCCCTGCTTTCCTACGCAAAGGCCTACGCGCACCTTCTCGGAACCCGTGACGTCGTGTTCGGTCAAGTCTTAGCCGGCCGA
ACACTGCCTCACCCTGAGGCAGACCGTACATTAGGACCATTATTCAACACTGTCGCGCAACGAATAACGCTTGATCCCACATTTATGAGTAACTGCGCTCTTGCGCAACGGCTCCAAA
GGGATGGCGTCGAAGCACAAAGACATCAGCATGCACCGCTGAGAATTATTCAGAATACGCTGCGTCAAGAGGGAAATCTTGACTCGAAGCAGTTGTTTGATGCACTCTTTGTGTTCCA
GAAGAGTGCCGCGCTCTCTCAGGGTATCCTGAACGAGCAGGAGATTTGGACATCATATGAAGATGAGGATTTCGTCGTTGACGCGGAATATAAACTCAATGTCGAGGTTGACCATTCT
CACGACGGGGTCATTGTTAGGGCTACGGCAAATGGTGCCTACCTTAACCAGAAAATGCTTGAGAGTTTCCTCAGTCAATACGTCGAGGTTTTCTGTGATGTCGTCGAGCATCCCGCAA
GATGTGTCACGGCGGTACCGCAGGGACTGGGAGGACTGCCGTTAGAACGGGCGAGTTCGAGATTCGGGCAACCGGTGACTGACGGCTCGAAGCCGGATTCTGCGCCCTCTACGCC
TGTGCCAGTGCATGAGGAAACCATTCGATCTGTTCTAGCAGACGTCGTGGGAATCTCTACTGACGATATCAAGCCAACCACGAGCATTTTCAACCTCGGGCTTGACTCTCTTTCAGCT
ATCAGGCTCGCTTCTCTTTGCCGCACAAAGGGTTTGAAAGTCAGTGTTGGGGATATTCTTCAGGGGAATACGCTGCGCGGTATAAGTACGCGTGTGCAGTTAGAATCCGAAGTCTCTA
CCACGACAAACGGGCACAACCCAGTTCATGGAACTTCGTCCTTGATTAAGGATTATCCTCACGTCGAACAAACCGTCATCTCTAATCTGCATCTCAGCAAAGAAGAGATCGAAACAATT
ATCCCCGTTCTCCCTGGCCAATTTCACCACCTGGTTGGCTGGCTCAAGTCAGACCGGAAACTGTTCGAAGCACCCTGGGCTTTTGTCGCTCGTGATGATAAGCGTATCAATGCCGATA
AGCTCCAGTGTGCGTGGGCCGATCTTCGCAAGCGACATCCTGTGCTACGGACGGCCTTTGCAGCTACCTCGGACTCAGAGGCTGTGCAGATTGTTCTGAGAACACCTGCAGAAAAC
TCGGATGCATTCAGAGTCATCGAATCTGCGGACAATATTGCCGACCTAGCCAGAGCGCACGCCCGCGAGGAAGCGTTGCACCCATCCTCCCTCTCTTCCCCGCCTGTGCGATTACG
GCACCTCAAGGCTGCGGATAGGGATGGCATCCTGCTTATTATCCATGCCTCTCTGTACGATGCATGGAGCATTCCGATGCTTGTCTCTGAACTTGGTAAACTCTACGACGATCAGCCA
ACAGATTTTACCACAGCCCCAGACTTCCCTGCCCTCGTGGACTTTTCACTTCGTGCTCTCTCCAACCTCGATGTCAACGAGAAGGATTACTGGACCTCGACTCTTAAGCCCGCTACAC
CGACTCTTGTCAGGAGCGCCGGTACGCAGAAATCAATTGCAAACAACGAACAGTTGTTCGTTGGCGAATGGGAAAGAGTCTCCAATCTATCTACGATGGAGAAAATATGTCGATCCGC
GGGATTCAGCCTTCAGACGATCATCCTCCTCGCTGTTGCGCGCTGCCTTGCGAGATCCACTGGCGTTGAGAGCCCCGTCATGGGACTCTATCAGAATGGGCGCTTGGCTGCGTTCG
ATGGAATTGAAAGGGTCCCGGGACCCTGTTTGAACGTCAATCCGTTTGTTGTGGAGGATGTGTTAACCAGTCTGGGCAATGAAGAGAAAGAGTGTGTCCTCAAGCAGGCGAGAAACA
TTCAGAGATCCCTCGCAGAACGGGTACCGTATGAGCAGAGTTCGCTTCGTAAAGTCCTAACATGGCTTAACCCGGAAAATGGAGAGGTAACACCGCTATTCAATATGTGGGTTAATCT
GCTTTGGATGCAGGACAGCACTTCCTCCACCCCCAGCCAGCAAGCCAATGACGAGAAAAGTGAAGCCGGGTTCTTCAAACCCCTTCGCATCGGCGTTCCGACAGACTTCATTCCTTC
TAAGCCGCTACCCCCATCATCTACATCGATAGATAGCCTCGATACATCTTACTTGCCAGACCAGAATATCTTCCTTGATATTGGTCCCGATCCAGCCACGGATAGTATTGGCTTTGGGG
TCCGTGTTGAGGGTGGCTTGCTGGCAGAGGGTGAGGTCAAAGAGCTTGTGGACGCGATTGCCGCTGAGATTGAAAGAGCTGTTGCTTGCTTAAAAGCTCACGTGCATCACCATCACC
ATCACTAA

**pHis$_{14}$-SidC NRPS (amino acid)**

MASHHHHHHHHTAMGKRKLAGSFRRYVATGLSRVSGAFRSRSASKERQSRGSGSLVERDTLAKDQ
GQGVCLQPAQVGAVPVTKVPALDTIEAYAGAFDKPIDGAGTCQRTSLVCSASSAVDGDVIDGLVQGY
ARFIAGLTGLDDIAFYSTRHEPFALEKSTSIIIASAASNELVCREVDTNHENDVQFYAELGRVEPENGR
REFQPNAFTLFIEPDSNRKKKNVLHLSFAYPRRLIPDAAVEQLLRTLLLHICESSPLKSPSTSQPELSIL
NFPPSMIPPTAQANGVENSTTNPHLLHSAFENWARKNPHFIALDFIHSLSSKTHRSEHSIITYAALDSA
ATNLAFHIRSLLRDSRKHGQIIPVHMPTSPELYISYLAVLKAGHAFCPIPQDVPARRIQEILSDIDAPIVLG
TSSKPPISAESSRSTSTWVNVTEVSKWRQMCGEQPADYSRPSLDHITIEQNQTAYLLFTSGSTGKPK
GVQISHLAASCSISSHATAIPLPGESPGNFRWFQFASPSFDPSLMEIFVTLSTGGTLCSADRRLTLANL
EATINESRATVMMATPSLATLLRPDRLETLEALWSMGEKLNRTVIDNFALDNVMNGDAETRRPRTLV
NAYGPTEGAINCTYYVAPFKRHMRGSIIGRPLPTCAMFILSPDSQVPVLVPTGTVGELAIGGPQVSKGY
LNLPEVTARVFIRSKEFGPLYRTGDKARIVWDESGHQVIEYLGRIRTDQVKINGRRVELGEIESVVAAV
EGVREAVAVVVKRDSKSNGGEQIVACLVVDAADGEGREKIAQQAKKNAAQHLASFMCPTTYTFFDVL
PRSSSGKVDRKALAVQLQEKPDVPIKNGLSEESAECWRHSDEAASSVQQLVIRLVAETGDVSDSAIK
PGTELYSVGIDSLGAMRFLQKLRDNGVHGLSVGEVLQTHTCQRLVSLVQSMLTNQNGEPNGILQKG
SITNDLQLRLQSFGRRYRMFCAESLDVPADTIHEVLPTTATQSGMLTSFLRSSAEQSYEKPTYIYHTVL
PLEPRTNIEKLKKAWFDVISNYDSFRTVFCMVDDELAPFAQCILTAEGVSSRDWNVYTSPNECSAEN
DIIDHALRSAEESITLRRPPWKLSLVQSSTKSIMILSMFHGIFDGGSLQLLLQDVSSAYSGNTLPQRTSL
THIVKHHFQADHTSTSKFWREYLQGYSLLPFPSLTPHRAPAQKSTGCAEVTSQLSYGALKKLSKSIGS
TPLSVLQAAWGAVVLSYTATPDQDVVLGSVMSGRLDPDSKDCIGPTFTITPSRISVQQLKGSGSLTNQ
SVVHYLSSSNAKALSHLQPQLGSVTVNGKVPYDTVLAYQDFDTDMKPSQTWSSVQHPAMANDFSV
MIEVVPNPDSTLTLRASFDTKLDSTGAQIMLKQMDDIVSYILNHPDSSFEDAPLQASLALKSKANPSPIT
APEVSEGALLQSQFEDHALSHPNDPALVFKQDLDNDDHPGNITWTYAQLNAMAEALAEHLLQVCGD
LRDASVPICIEKSPPLYVAILGILKAGGAWCPIDTLSPAQRRHDLIARTSAGILLVSGLDTPQPQNAVPA
GVRVIDVSKFIQNVSSDNTPQSSRHRATPRNTAYLIWTSGTTGAPKGVPITHSAAVSSMRSLQTDIPG
NEDGSPIRCLQFSQPTFDVSIQDLFYTWGLGGALISGTREIMLESFPKLANITKATHAHLTPAFAAGVA
RKSCKTLKVVTMIGEKLTQSVADDWGTDMRAFNTYGPAEATVVSTIREFGNEHRSVKSANIGWPMS
SVSVFVMSKDKRVLMKNAIGELALGGPQLSPEYLNLKDVTDTKYIWNEDAGQRLYYTGDLVRMLSDG
SLEYITRVDDLVKLGGIRIELSEISFALRGCHELVESVETMILSRKDRPVRVVVAFLCAPKAAGDADEGL
LVLDDTGRDIARAASLQARNVLPENMIPSVYLIVKKIPKTPSAKVDRRALQAAYAAVDIDKWENNVNPE
GPGDADEDDAATATQIIETVAALVHVESSTITKSNRLRSLGVDSLCATRLAFRLKEAGFGLSVMDVLAC
TTIQDLVRLAQSTSFSSSANSASPHNDKFDIVSFNKIWHTLVAAAAKIPEKDAFTTIRATAIQESLLTETM
GTYDMYWSNHFFRLDRSVDIPRLRQAWYAVCQKTETLRTGFIPVAQTEAKNKKQAKDSGFSILQVVY
KLPAVDWEAHTYRKEEWSWVLKNRVRDIMTAHQKNYFCHPPWAVTILEEGTERVMVLTLHHSIHDE
PSLKFLMDDVRAAYTYKPPLRTQLTPALALVLPTPSKYAEAVDFWSLELKPYAALDVPVWPDLTGKRV

SPGAAPEYKLISEAMSITSSFPVLEKVAADLGLKSVASIIRAAWAFVSLSYLGLSGTVFAETLSDRVFDP
SLESAVGPFISVVPVPFRVEGDTTVRKILAEQHRLSLQSWKHRHVHARDIRKALKRQRGEPLYPAVFN
FHALDESKDRKIASLPGLWHELEDQIGLHVEHPMAMNVFQSPSGNMTLEASSDSRIFSREHLRLFVR
QIDALISEMLLSPDESLSGLVNRLPSDLRSLSNRIVSHEVANSIHQAPTYWLEKFADTHPHWTAVEVAS
SISTNGIEKEAMSYGSLNSAANRVAAYLASFRYKNRVVGVCAGRTLASYPIIIGIFKSGNTYLPIDESLP
ADRKAFLLEDAKCPVVFTELGLRNSFAGAPDTCRVECIDDPALQRSLDEMPSTNKDYSSHPDDVSYL
LFTSGSTGKPKGVMVTRANLSSFIESISEFACRIAPDTLKLGGTGRYLAQANRAFDPHLLEMFFPWRH
GMATVTAPRPMILDDIGTTLSKWSITHASFVPSLVDQSDITPQQCPNLRFMTVGGEKITQKVLDTWAS
APNVAIVNAYGPTEVTIGCTFAHINPSTNLRNIGPPLTACTAHVLIPGTMKYALRGQTGELCFSGDLVA
RGYLNRPDATAANFITGPNGAKMYRTGDIGRLMSDDSVEYLGRGDDQTKIRGQRLELGEVSEVLRAS
SPVAVDIVTTVAKHPDLGKVQLITFVSRAKKRTVNEEVQFLFSDFGTLGQELRDICAKKLPAYMVPDLI
LPVTSIPVAAMSGKADMKVLQKLFTELPLQVVLQGNNAIANGTGSERPLNPDEIAVVGEICQVISADSH
SFSPMTNIFEIGIDSLSAIGLSVRLRGIGYAASVAAIMANPVVEQLARLPRASEHGVNDHADYFAQRCK
ELESQYRSVFRDAVEVAVVRPCLPLQEGLIARSMNSNSGDGKLYVNHVILQLNKDVDTRKLKSSWED
VAKENEILRTAFAPLEKEIAQVVLSNASYQMQWTEGKYEDLDEAIQARNEKQGQISRLIIADLSTVPPA
RFHLASSASGKPLALFISIHHGLYDGESFAMMLDEVAARYEGRKVGERGSPSVFLRHVCSQDTEEAK
RHWMQQLSGCTPTIFRANGNAIKNTISIRRTGNAKLSDLETRSSTLQTTVPNLLQAVFALLLADHTGIF
DVTYGLVLSGRTISAPGADSVLLPAITTIPGRLNMSDLKTVNDVVKVVQRATARSLDFQHTPLRKIQQ
WLKSEAPLFDCLFSYIRATAPPGHNLWAELDSHMPSEYPLALEVQADNAANTLKLECIFSSDFGPRQV
GEEFLEKMDAVISEVVSGSSLPLENFNTVRSATSASHGASVQWDESSWTSSESRIREITATFCGLNVE
AVSKGASFFSLGIDSVTALQFARRLRDEGFKVSSSEIMRFSCVGSLTGHIESSSALQTNGIGKTETAISI
ETYAKHIPLLGKNDSITSLFECTPLQSGMITQTISSGGKVYINPHPIRLRDNVKVEKLKEALRHVVQANE
ILRTSFHLIPDLGESWIGAVHEEPKFEWSEINMPSGANALSEVMNLYTFCEEASFERPPIRSGLVNQP
GYRILIAVLHHSLYDGAYLPFVFEDLATIYAGGTLAQRPQFSEMVPYMLSGQDESCAFWVDRLRDYVP
VEICPLPKLDSTPNMLTAKNRIPLPLHSITESCKTMSVTIQTVSLLSYAKAYAHLLGTRDVVFGQVLAGR
TLPHPEADRTLGPLFNTVAQRITLDPTFMSNCALAQRLQRDGVEAQRHQHAPLRIIQNTLRQEGNLDS
KQLFDALFVFQKSAALSQGILNEQEIWTSYEDEDFVVDAEYKLNVEVDHSHDGVIVRATANGAYLNQK
MLESFLSQYVEVFCDVVEHPARCVTAVPQGLGGLPLERASSRFGQPVTDGSKPDSAPSTPVPVHEE
TIRSVLADVVGISTDDIKPTTSIFNLGLDSLSAIRLASLCRTKGLKVSVGDILQGNTLRGISTRVQLESEV
STTTNGHNPVHGTSSLIKDYPHVEQTVISNLHLSKEEIETIIPVLPGQFHHLVGWLKSDRKLFEAPWAF
VARDDKRINADKLQCAWADLRKRHPVLRTAFAATSDSEAVQIVLRTPAENSDAFRVIESADNIADLAR
AHAREEALHPSSLSSPPVRLRHLKAADRDGILLIIHASLYDAWSIPMLVSELGKLYDDQPTDFTTAPDF
PALVDFSLRALSNLDVNEKDYWTSTLKPATPTLVRSAGTQKSIANNEQLFVGEWERVSNLSTMEKICR
SAGFSLQTIILLAVARCLARSTGVESPVMGLYQNGRLAAFDGIERVPGPCLNVNPFVVEDVLTSLGNE
EKECVLKQARNIQRSLAERVPYEQSSLRKVLTWLNPENGEVTPLFNMWVNLLWMQDSTSSTPSQQA
NDEKSEAGFFKPLRIGVPTDFIPSKPLPPSSTSIDSLDTSYLPDQNIFLDIGPDPATDSIGFGVRVEGGL
LAEGEVKELVDAIAAEIERAVACLKAHVHHHHHH
