# Supplementary material for: Elucidating the molecular programming of a nonlinear non-ribosomal peptide synthetase responsible for fungal siderophore biosynthesis
Source: Nat Commun. 2023 May 17;14:2832. doi: 10.1038/s41467-023-38484-8 (PMC10192304; doi:10.1038/s41467-023-38484-8)
Supplement: Supplementary file 5 — Reporting Summary [file 41467_2023_38484_MOESM5_ESM.pdf]

nature portfolio

Corresponding author(s): Dr. Matthew Jenner
Dr. Yang Hai

Last updated by author(s): 04/04/2023

Reporting Summary

Nature Portfolio wishes to improve the reproducibility of the work that we publish. This form provides structure for consistency and transparency in reporting. For further information on Nature Portfolio policies, see our Editorial Policies and the Editorial Policy Checklist.

Statistics

For all statistical analyses, confirm that the following items are present in the figure legend, table legend, main text, or Methods section.

n/a	Confirmed	
x	☐	The exact sample size (n) for each experimental group/condition, given as a discrete number and unit of measurement
x	☐	A statement on whether measurements were taken from distinct samples or whether the same sample was measured repeatedly
x	☐	The statistical test(s) used AND whether they are one- or two-sided<br>*Only common tests should be described solely by name; describe more complex techniques in the Methods section.*
x	☐	A description of all covariates tested
x	☐	A description of any assumptions or corrections, such as tests of normality and adjustment for multiple comparisons
x	☐	A full description of the statistical parameters including central tendency (e.g. means) or other basic estimates (e.g. regression coefficient) AND variation (e.g. standard deviation) or associated estimates of uncertainty (e.g. confidence intervals)
x	☐	For null hypothesis testing, the test statistic (e.g. F, t, r) with confidence intervals, effect sizes, degrees of freedom and P value noted<br>*Give P values as exact values whenever suitable.*
x	☐	For Bayesian analysis, information on the choice of priors and Markov chain Monte Carlo settings
x	☐	For hierarchical and complex designs, identification of the appropriate level for tests and full reporting of outcomes
x	☐	Estimates of effect sizes (e.g. Cohen's d, Pearson's r), indicating how they were calculated

Our web collection on statistics for biologists contains articles on many of the points above.

Software and code

Policy information about availability of computer code

Data collection	Intact protein mass spectrometry: Bruker otof control 4.0; LC-MS Analyses of enzymatic reactions: Shimadzu LabSolutions; Generation of structural models for SidC: ColabFold v1.5.2.
Data analysis	Bruker Compass DataAnalysis 4.4, Shimadzu LabSolutions, PyMOL v1.3, AlphaFold (ColabFold notebook (v1.5.2)).

For manuscripts utilizing custom algorithms or software that are central to the research but not yet described in published literature, software must be made available to editors and reviewers. We strongly encourage code deposition in a community repository (e.g. GitHub). See the Nature Portfolio guidelines for submitting code & software for further information.

Data

Policy information about availability of data

All manuscripts must include a data availability statement. This statement should provide the following information, where applicable:

- Accession codes, unique identifiers, or web links for publicly available datasets
- A description of any restrictions on data availability
- For clinical datasets or third party data, please ensure that the statement adheres to our policy

Authors confirm that all relevant data are included in the paper and/or its supplementary information. Data Availability Statement (as appears in manuscript): The minimum dataset necessary to interpret, verify and extend the work is provided in the manuscript and supplementary information. The raw data for Figures 2 and 3, and Supplementary Figures 11, 12 and 16, which were processed via standard deconvolution, are available upon written request to the corresponding authors. GenBank accessions have been provided for SidC (XM_653119, [https://www.ncbi.nlm.nih.gov/nuccore/XM_658335.1]), SidA (XM_658335, [https://

www.ncbi.nlm.nih.gov/nuccore/XM_658335.1] and SidL (XM_652967, [https://www.ncbi.nlm.nih.gov/nuccore/XM_652967]) in the methods section. The DNA and amino acid sequence of the full-length SidC construct used in this study is reported in the supplementary information. Co-ordinate files for structural models of SidC have been deposited in Mendeley Data DOI: 10.17632/c3ymyp3yx4.1. A reporting summary for this Article is available as a supplementary information file.

Human research participants

Policy information about studies involving human research participants and Sex and Gender in Research.

Reporting on sex and gender	N/A.
Population characteristics	N/A.
Recruitment	N/A.
Ethics oversight	N/A.

Note that full information on the approval of the study protocol must also be provided in the manuscript.

Field-specific reporting

Please select the one below that is the best fit for your research. If you are not sure, read the appropriate sections before making your selection.

- [x] Life sciences
- [ ] Behavioural & social sciences
- [ ] Ecological, evolutionary & environmental sciences

For a reference copy of the document with all sections, see nature.com/documents/nr-reporting-summary-flat.pdf

Life sciences study design

All studies must disclose on these points even when the disclosure is negative.

Sample size	No sample size calculations were performed as data processing did not involve statistical analysis of large datasets / samples. Intact protein mass spectrometry experiments were conducted in duplicate and LC-MS analyses in triplicate, which is sufficient for qualitative assessment of reproducibility by comparison of spectra / chromatograms.
Data exclusions	No data were excluded from analyses.
Replication	LC-MS analyses of enzymatic reactions for metabolite production were conducted in triplicate - with all attempts successful. Intact protein mass spectrometry experiments were conducted in duplicate. Protein expression constructs / mutants were confirmed by DNA sequencing.
Randomization	N/A - no experimental groups.
Blinding	N/A - no experimental groups.

Reporting for specific materials, systems and methods

We require information from authors about some types of materials, experimental systems and methods used in many studies. Here, indicate whether each material, system or method listed is relevant to your study. If you are not sure if a list item applies to your research, read the appropriate section before selecting a response.

Materials & experimental systems

n/a	Involved in the study	
x		Antibodies
x		Eukaryotic cell lines
x		Palaeontology and archaeology
x		Animals and other organisms
x		Clinical data
x		Dual use research of concern

Methods

n/a	Involved in the study	
x		ChIP-seq
x		Flow cytometry
x		MRI-based neuroimaging
